# DNA-based molecular classifiers for the profiling of gene expression signatures

**DOI:** 10.1186/s12951-024-02445-0

**Published:** 2024-04-17

**Authors:** Li Zhang, Qian Liu, Yongcan Guo, Luyao Tian, Kena Chen, Dan Bai, Hongyan Yu, Xiaole Han, Wang Luo, Tong Feng, Shixiong Deng, Guoming Xie

**Affiliations:** 1 Key Laboratory of Laboratory Medical Diagnostics, Ministry of Education, Department of Laboratory Medicine, Chongqing Medical University, Chongqing, 400016 China; 2 Department of Forensic Medicine, Chongqing Medical University, Chongqing, 400016 China; 3 Nuclear Medicine Department, The Second Affiliated Hospital of Chongqing Medical University, Chongqing, 400010 China; 4 Clinical Laboratory, Traditional Chinese Medicine Hospital Affiliated to Southwest Medical University, Luzhou, 646000 China

## Abstract

Although gene expression signatures offer tremendous potential in diseases diagnostic and prognostic, but massive gene expression signatures caused challenges for experimental detection and computational analysis in clinical setting. Here, we introduce a universal DNA-based molecular classifier for profiling gene expression signatures and generating immediate diagnostic outcomes. The molecular classifier begins with feature transformation, a modular and programmable strategy was used to capture relative relationships of low-concentration RNAs and convert them to general coding inputs. Then, competitive inhibition of the DNA catalytic reaction enables strict weight assignment for different inputs according to their importance, followed by summation, annihilation and reporting to accurately implement the mathematical model of the classifier. We validated the entire workflow by utilizing miRNA expression levels for the diagnosis of hepatocellular carcinoma (HCC) in clinical samples with an accuracy 85.7%. The results demonstrate the molecular classifier provides a universal solution to explore the correlation between gene expression patterns and disease diagnostics, monitoring, and prognosis, and supports personalized healthcare in primary care.

## Inroduction

Gene expression signatures hold the key to understanding various diseases and open the door to patient-specific personalized medicine [1–5]. With the rapid advancement of techniques for the quantification of gene expression, such as quantitative reverse transcription PCR (RT-qPCR), microarrays and RNA sequencing, the identification of gene expression signatures allows clinicians to better evaluate patient conditions, predict patient prognosis, and optimize clinical diagnosis and treatment plans [6, 7]. However, the time of clinicians, who are expected to utilize these signatures, is too precious to be wasted in evaluating the relevance and significance of gene expression signatures. A promising direction is involvs the analysis and identification of gene expression signatures by scientists to ensure that robust and comprehensible results can be delivered to clinicians [8, 9]. In addition, current profiling techniques are generally requires costly equipment, lengthy protocols, skilled technicians, and complex bioanalysis pipelines to accurately quantify each of these markers independently and interpret the result. It is necessary to develop a flexible, interpretable, and accurate approach to enable profiling of gene expression signatures in low-resource conditions.

Over the past few decades, with progress in sequencing methods and information analysis, a growing number of coding or non-coding RNAs have been identified, and many of them exhibit highly tissue-specific expression patterns and play crucial roles in biological processes associated with diseases [10–13]. The expression profiles of these RNAs exhibit great potential to serve as noninvasive biomarkers for the diagnosis, progression, and prognosis of cancers and other diseases. For instance, a set of three genes (GBP5, DUSP3, and KLF2) signature in whole blood was identified to discriminate active tuberculosis from latent tuberculosis (global area under the ROC curve (AUC) 0.88 [95$\%$ CI 0.84–0.92]) [14]. Furthermore, hundreds of studies have proved that circulating miRNAs are potential ideal biomarkers for treatment management, and a substantial increase in the number of clinical trials focused on different cancer types and disease stages also indicates the ongoing trends [15–17]. Nevertheless, new technologies generate increasingly large databases that will be more and more difficult to analyze, gene expression signatures remain a certain distance from clinical practice [8]. Further standardization and improvement of current technologies potentially promote this process [18]. Moreover, the application of machine learning and new technologies for fast and robust profiling methods at the point of care could ensure effective transition of gene expression signatures into clinics, thus substantially improving patient management and outcome [19, 20].

With the inherent stability, flexibility, and programmability of DNA, DNA nanotechnology is perfectly fitted for building molecular classifiers to integrate multiple chemical inputs into a low dimensional output (e.g., “healthy”/“disease”). Recent advances in DNA-based molecular computation have demonstrated the feasibility and validity of interpretable molecular profiling methods [21–26]. For example, Seelig and coworkers designed a molecular multi-gene classifier for analysing gene expression signatures, and the classification results were interpreted by the corresponding fluorescent signals [21]. Similarly, Fan and coworkers introduced a DNA-encoded molecular classifier to process multidimensional molecular clinical data, classes of molecules were translated to unified electrochemical sensor signals [25]. These studies implemented proof-of-concept of machine learning algorithms, such as logistic regression and support vector machine (SVM), in the application of interpretable classifiers by assigning a numerical weight to the target molecule to capture its importance. However, most of the current DNA computation systems include the discrete integers as weights in diagnostic applications, which are not well matched to continuous optimization process of machine learning and result in a loss of accuracy [27]. Furthermore, input sequences can be highly heterogeneous, intricately designed DNA circuits are tailored to specific sequences, as their performance is influenced by thermodynamics and dynamics [28–30]. To realize the application and expansion of DNA computing in personalized medicine, it is thus necessary to explore a universal molecular classification workflow that processes various inputs and generates accurate and intelligible results [31].

**Fig. 1:**
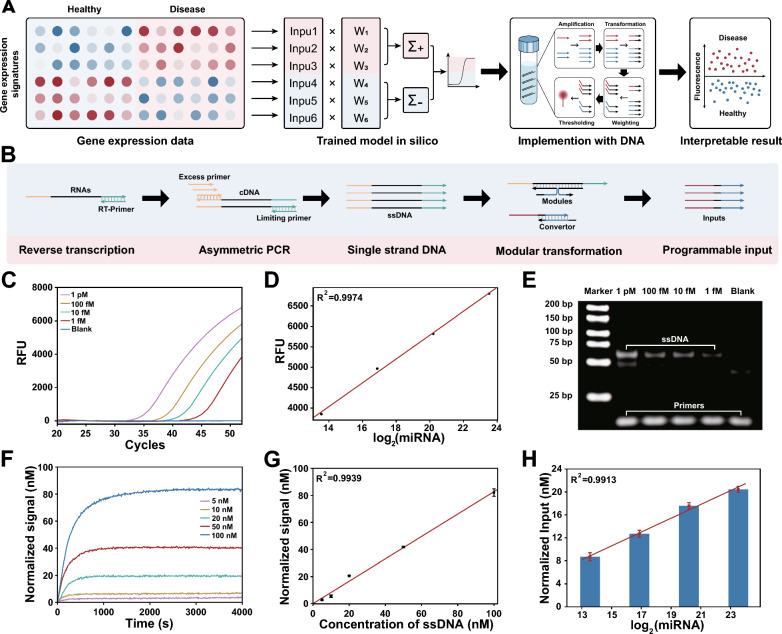
DNA-based molecular classifier for profiling of gene expression signatures. **A** Universal workflow for DNA-based molecular classifier to interpretation of gene expression signatures. **B** Process of asymmetric PCR and associative strand displacement to capture relative relationship of low-concentration RNAs and convert them to general coding input. **C** Asymmetric PCR for different concentrations of miRNAs. **D** Plot of fluorescence at cycle 52 versus logarithm base 2 of initial miRNA concentrations. $R^2$ = 0.9974. **E** Native PAGE performed to analyze PCR products derived from different initial concentrations of miRNA. **F** Fluorescence kinetics experiments for various concentrations of ssDNA are transformed into programmable inputs. **G** Plots of Steady-state fluorescence intensity versus initial ssDNA concentrations. $R^2$ = 0.9938. **H** miRNAs are transformed into programmable inputs by asymmetric PCR and associative strand displacement. As expected, we observed a linear relationship between logarithm base 2 of initial miRNA concentrations and concentration of input. $R^2$ = 0.9913

Herein, a general workflow was constructed based on a DNA-based molecular classifier for the interpretation of gene expression signatures (Fig. 1A). To ensure universality of the molecular classifier, we first designed a highly modular and programmable transformation strategy to capture the relative relationships of low-concentration RNAs and convert them to general coding inputs. Next, we demonstrate the precision of multi-input line classifier. Competitive inhibition of DNA catalytic reaction enables strict weight assignment for different inputs, followed by summation, annihilation and reporting as winner-take-all game to experimentally implement the mathematical model of classifier [32]. Based on the above, we validated the entire workflow by utilizing the miRNA expression levels for the diagnosis of hepatocellular carcinoma (HCC). Publicly available serum miRNA profile data corresponding to 345 HCC and 958 healthy individuals from Gene Expression Omnibus (GEO) were used to construct a linear classifier in silico. The trained classifier is subsequently decoded into transformational and computational circuits at the molecular level. Finally, synthetic and clinical samples were used to verify the performance of our workflow in gene expression profiling.

## Experiment section

### DNA and RNA oligonucleotides

All DNA and RNA oligonucleotides were synthesized and purified by Sangon Biotech (Shanghai) Co., Ltd. All DNA and RNA sequences are listed in Additional file 1: Table S1-3. Individual DNA was suspended to 100 µM in 1× TE buffer (10 mM Tris-HCl, 1 mM EDTA, pH 8.0). RNAs were stored in RNase-free $\mathrm {ddH_{2}O}$ at − 80 ^∘^C until needed.

### DNA probe preparation for molecular classifier

Single-stranded species were diluted to 10 µM in 1× TE buffer with 12.5 mM $\mathrm {Mg^{2+}}$ . DNA probes (converters, competitive inhibition systems and annihilators) consist of two or three distinct strands were mixed stoichiometrically with 20% excess of the target binding strand, and thermally annealed by heating to 95 ^∘^C for 1 min, followed by cooling from 95 to 25 ^∘^C over the course of 60 min (Bio-rad T100). Annealed probes were purified with 12% non-denaturing PAGE gel. Gel bands were visualized using ultraviolet light, and then cut out and extracted by a spin column PAGE gel DNA extraction kit (Sangon Biotech, B610357). Finally, purified probes were eluted into $1 \times$ TE buffer with 12.5 mM $\mathrm {Mg^{2+}}$ .

### RNA extraction, reverse transcription and asymmetric PCR

miRNAs in plasma samples were extracted using a plasma miRNA isolation kit (TIANGEN Biotech, DP503) according to the manufacturer’s instructions. The isolated miRNAs were stored in nuclease-free water at − 80 ^∘^C until needed. Synthetic or extracted miRNAs were first reverse transcribed into first strand cDNA using a miRNA first strand cDNA synthesis kit (Tailing Reaction) (Sangon Biotech, B432451). Reverse transcription was carried out with a total 20 µL volume containing 10 µL $2\times$ miRNA P-RT Solution mix, 2 µL miRNA P-RT Enzyme mix, 100 ng Extracted miRNAs, and add RNase-free water to 20 µL. The reverse transcription reactions were kept at 37 ^∘^C for 60 min, and then at 85 ^∘^C for 5 min on a Bio-rad CFX96 system. Asymmetric PCR was performed in a 20 µL system including 10 µL $2\times$ AceQ qPCR Probe Master Mix (Vazyme, Q112), 2 µL cDNA, 1 µM excess primer (specific to miRNAs), 25 nM limiting primer (universal primer), 200 nM Taqman probe (if needed), and add RNase-free water to 20 µL. The suitable cycling condition was 95 ^∘^C for 5 min, 10 cycles of 95 ^∘^C for 10 s and 55 ^∘^C for 30 s, followed by 42 cycles of 10 s at 95 ^∘^C and 30 s at 50 ^∘^C.

### SVM training and validation in silico

To build a SVM model for molecular classifier, the classification problem was simplified by distinguishing only between HCC and healthy individuals. A publicly available serum microRNA profiles data (NCBI GSE113740) corresponding to 345 HCC and 958 healthy individuals were used for classifier training. Firstly, differential expression analysis was used to identify miRNAs that were differentially expressed between cancer and healthy groups. Then, a random-forest based algorithm was applied to assess the relevance of each signature by ranking them based on their predictive importance. We subsequently trained an SVM classifier (with a linear kernel) consisting of 1 to 10 prominently ranked miRNAs. Finally, we selected the classifier with the highest AUC value for experimental implementation. Please refer to Additional file 1: Text S4 for detailed processes.

### Fluorescence kinetic measurements

Fluorescence kinetics data were collected by using a Cary Eclipse Fluorescence Spectrophotometer (Agilent) for single measurements and a Rotor-Gene Q (QIAGEN) for high-throughput measurements. All the measurements were repeated at least three times. The reactions were carried out in $1 \times$ TE buffer with 12.5 mM $\mathrm {Mg^{2+}}$.

### Fluorescence normalization

Arbitrary fluorescence units were normalized to concentrations using a standard curve of each reporter complex. To establish the standard curve, the annealed reporter complex was suspended in $1 \times$ TE buffer with 12.5 mM $\mathrm {Mg^{2+}}$, and an initial baseline fluorescence signal was measured. Subsequently, a range of known concentrations of reporter initiator strands were added. The steady-state fluorescence of various reporter concentrations was utilized to construct a linear standard curve (Additional file 1: Fig. S11).

## Results and discussion

### Modular and programmable transformation of signatures

The substantial heterogeneity and intricate secondary structures of RNAs significantly restrict the commonality of DNA-based computation in gene expression signature profiling. Moreover, RNAs are typically found at concentrations ranging from attomolar to femtomolar in tissue and blood samples, necessitating a pre-amplification step for observable computation reactions. Herein, we developed a strategy based on asymmetric PCR and associative strand displacement, to modularly amplify and transform gene expression signatures into programmable inputs (Fig. 1B).

Asymmetric PCR was employed to achieve nearly linear amplification of RNAs, relative to their logarithmic initial concentrations. Using miRNA-21 as an example, we first employed a commercial kit that enables simultaneous poly(A) tailing reaction and reverse transcription to generate first strand cDNA. Subsequently, the generated cDNA was amplified by a specific primer and a universal primer, where the specific primer acts as an excess primer and the universal primer functions as a limiting primer. By adjusting the melting temperature (Tm) and stoichiometric ratio of the limiting and excess primer, the initial exponential phase of the reaction generates double-stranded amplicons until the limiting primers are exhausted, and the reaction switches to synthesis of only excess primer single strand DNA (ssDNA) [33]. At a specific cycle number, the ratio of generated ssDNA is consistent with that of the logarithmic initial concentrations of the RNAs (details of the proof process are provided in Additional file 1: Text S1). Fig. 1C–E showed the generated ssDNA from a series of initial miRNA concentrations ranging from 0.1 to 10 pM. The results demonstrated a linear relationship between the initial logarithmic concentrations of the miRNAs and the ssDNA produced by asymmetric PCR, confirming the feasibility of this method for subsequent molecular classifier.

Next, we designed associative strand displacement to modularly decouple sequence constraints between RNAs and subsequent DNA-based molecular classifiers. As shown in Fig. 1B, the two splitting modules partially complement to the generated ssDNA, and the remaining parts form a complete strand to trigger following strand displacement. Through the process of associative strand displacement, heterogeneous RNAs were transformed into a programmable sequence for universality. We first investigated the effect of hybridization length with ssDNA on the yield of strand replacement, each module was designed with at least 13 bases complementary to ssDNA to ensure a high yield (Additional file 1: Fig. S12). In addition, the split position and length of junction were optimized to minimize leakage during the process of conversion. According to the results shown in Additional file 1: Figs. S13 and S14, we strategically placed the split position 4 nt away from the toehold region and eliminated junction between two modules. Under optimal conditions, ssDNA were efficiently translated to programmable input for subsequent molecular classification (Fig. 1F, G). In general, these processing steps transform signatures into programmable inputs while preserving their original concentration relationship (Fig. 1H).

**Fig. 2:**
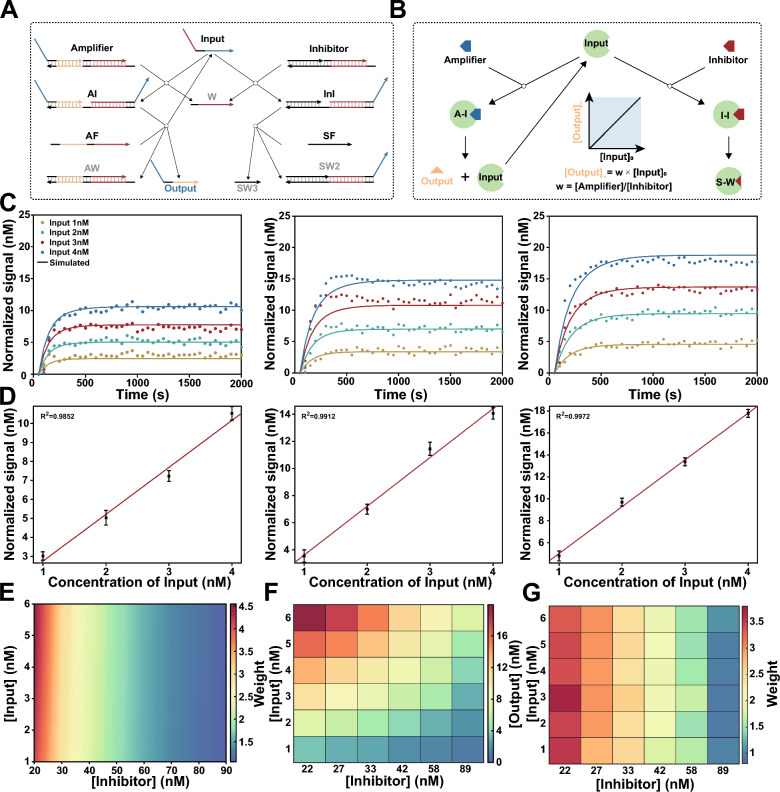
Arbitrary weight assignment to signatures with competitive inhibition system. **A** Scheme for DNA-based competitive inhibition system. DNA non-covalent catalysis adapted from entropy-driven reactions. Input act as a catalyst to catalyze the reaction between *Amplifier* and AF, resulting in the production of output for the following computation. Meanwhile, *Input* can also participate in *Inhibitor* to generate inactive products *Waste* with a same rate constant. **B** Scheme for irreversible competitive inhibition model of enzymatic reactions. when $[Input]_{0} \le [Inhibitor]_{0}$ , final concentration of *Output* is linear in the initial concentration of *Input*. **C** Fluorescence kinetics of different concentrations of *Input* (1, 2, 3 and 4 nM) with different weights (w = 2.5, 3.5, and 4.5). Amplifier = 100 nM. Inhibitor = 38.5 nM (w = 2.5), 27.5 nM (w = 3.5), 21.5 nM (w = 4.5). Experiment in scatter plot. Simulation in solid lines. **D** The linear relationships between the concentration of *Input* and *Output* corresponding to w = 2.5, 3.5, and 4.5. **E** Simulation results show different weights can be achieved by adjusting the concentration of *Inhibitor* (20 to 89 nM), and it was observed that the performance remained consistent across various input concentrations (1 to 6 nM). **F** Experimental validation for different concentrations of inhibitors, *Output* values correspond to different *Input* values. **G** Experimental demonstrated precise weighting of input by adjusting the concentration of *Inhibitor*.

### Arbitrary weight assignment to signatures

In molecular classifiers, various gene expression signatures hold their contributions to state of disease respectively, and a corresponding numerical weight is assigned to each signature in the machine learning model in silico. To implement arbitrary weight assignment at the molecular level, we designed a DNA catalytic system with an inhibitor as shown in Fig. 2A. Similar to the irreversible competitive inhibition model of enzymatic reactions (Fig. 2B):

1 $$\begin{aligned} Input+Amplifier \xrightarrow {k}Input + Output \end{aligned}$$

2 $$\begin{aligned} Input+Inhibitor \xrightarrow {k} Waste \end{aligned}$$

In an ideal situation, the final concentration of Output can be computed by integrating the corresponding differential equations:

3 $$\begin{aligned} \lim \limits _{t\rightarrow \infty }[Output](t)=[Input]_{0} \frac{[Amplifier]_0}{[Inhibitor]_0} \end{aligned}$$

As a consequence, we can exactly weight signatures by adjusting the initial concentration of the *Amplifier* and *Inhibitor* (see Additional file 1: Text S2 for details).

To experimentally validate this strategy, we designed an entropy driven catalytic system, namely, *Amplifier*, and a cascade reaction as corresponding *Inhibitor* maintained a consistent reaction rate [34, 35]. *Amplifier* can be catalyzed by inputs and release output strands, which then interact with double-stranded fluorescent reporters to determine their concentration. We first implemented weights (W) = 2.5, 3.5 or 4.5 for a series of concentrations of input ([Input] = 1, 2, 3 or 4 nM). Kinetic fluorescence measurements were performed after adding inputs to the competitive inhibition system, and we found that the final signal was linearly proportional to the stoichiometric ratio of $Amplifier_0$ and $Inhibitor_0$ for all concentrations of the inputs (Fig. 2C, D). The relationship between concentration of input and normalized signal was fitted to the linear equation $[Signal] = W \times [Input]$, the coefficients of determination ($R^2$) were greater than 0.98 for all the weights.

To further demonstrate that this mechanism can be used to assign an arbitrary weight to varying concentrations of input, we simulated the competitive inhibition system using ordinary differential equations (ODEs) (see Additional file 1: Text S3 for details). As shown in Fig. 2E, different weights were achieved by adjusting the concentration of inhibitor, and the performance remained consistent across various input concentrations. Then, we experimentally verified the simulated results, and the concentrations of *Output* and weights corresponding to different input concentrations demonstrated the precise weighting of input by the DNA-based competitive inhibition system (Fig. 2F, G).

**Fig. 3:**
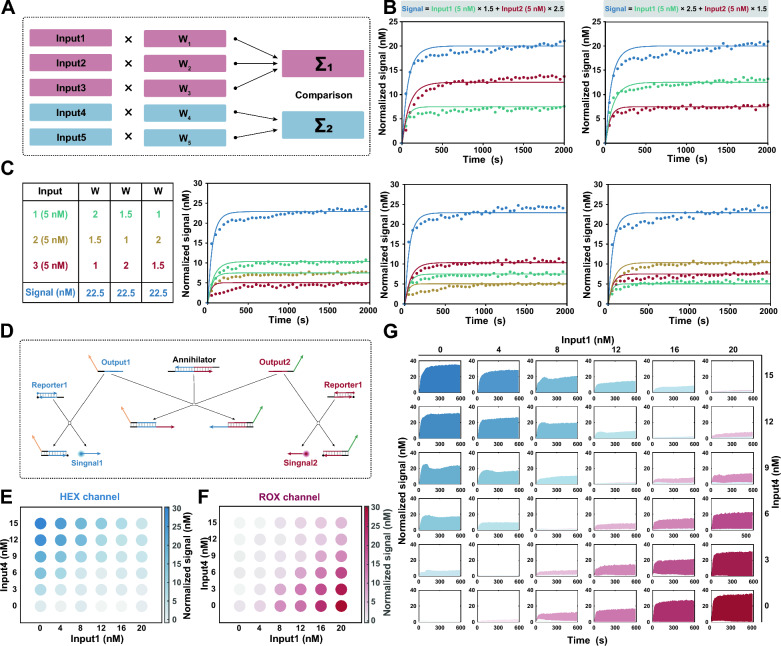
Implementation of mathematical operations for molecular classifier. **A** Scheme for a comprehensive linear classifier in silico. **B** Fluorescence kinetics for two-input summation systems. Amplifier = 100 nM. Inhibitor = 65.0 nM (w = 1.5), 38.5 nM (w = 2.5). Experiment in scatter plot. Simulation in solid lines. **C** Fluorescence kinetics for three-input summation systems. Amplifier = 100 nM. Inhibitor = 89.0 nM (w = 1), 65.0 nM (w = 1.5), 38.5 nM (w = 3.5). Experiment in scatter plot. Simulation in solid lines. **D** Annihilation reaction based on DNA cooperative hybridization mechanism enables comparison between the output strands. **E** Endpoint fluorescence measurements captured from the HEX channel for the linear classifier. **F** Endpoint fluorescence measurements captured from the ROX channel for the linear classifier. **G** Fluorescence kinetics captured from the HEX and ROX channel for the linear classifier. Blue for HEX channel. Red for ROX channel

### Mathematical operations for the molecular classifier

To construct a comprehensive linear classifier, it is essential to employ mathematical operations that sum the weights and compare the resulting summation to the predefined threshold value, thereby obtaining the desired logistic response [36] (Fig. 3A). In DNA computation, the process of arithmetic summation can be naturally implemented through the production of identical output strands. Herein, we designed output strands that contain the same domain, allowing them to react with fluorescent reporters, for each input. The final fluorescence signal thus indicates the summation of weighted inputs:

4 $$\begin{aligned} \lim \limits _{t\rightarrow \infty }[Signal](t)=\sum \limits _{i}W_i\times [Input_i] \end{aligned}$$

Two-input and three-input summation systems were designed to verify the summation of weighted inputs, and the response signals were found to be consistent with the results of mathematical calculations (Fig. 3B, C). Simultaneously, another class of inputs, which exhibit a negative correlation with the outcome, yielded outputs containing distinct sequences for the negative reporters. The concentrations of different output strands individually represent the cumulative contributions of positive and negative inputs.

Then, a comparison between the output strands was implemented to generate the final result. It is convenient to accomplish the comparison by an annihilation reaction, summed output strands for positive and negative inputs were consumed at a stoichiometric ratio of 1:1 (Additional file 1: Figs. S15, S16). We carried out the annihilation reaction based on DNA cooperative hybridization mechanism [37]. As shown in Fig. 3D, one of the output strands is reversibly incorporated into the annihilator through the binding of a toehold. In the presence of another output strand, two outputs and an annihilator will irreversibly collapse into two waste molecules. The annihilation efficiency is highly dependent on the length of the toehold on the annihilator, toeholds with sufficient length have been intentionally designed to ensure the complete consumption of all minority species. In practice, HEX and ROX labeled reporters are designed to report the corresponding outputs associated with positive and negative weights in our system. Annihilation reactions with series of output concentrations ranging from 0 to 50 nM illustrate the successful implementation of subtraction.

We experimentally tested the main mathematical operations of the molecular classifier. Taking a simple linear classifier $[Signal]=1.5\times [Input1]-2\times [Input4]$ as an example, we combined a range of concentrations of each input to characterize the response. The fluorescence signals of 36 various input combinations were recorded fluorescence signal after they were added to the corresponding molecular computing system. Fig. 3E, F illustrate the endpoint fluorescence measurements captured from the HEX and ROX channels. Notably, a significant increase in fluorescence was observed in the HEX channel only when the value of weighted $Input_1$ surpassed that of $Input_4$, while no fluorescence signal was detected in the ROX channel, and vice versa. Among the experiments for which the weighted input was the same, both fluorescent signals were low (Fig. 3G), and were located on the diagonal. These observations suggest that the proposed design has credible mathematical operations.

**Fig. 4:**
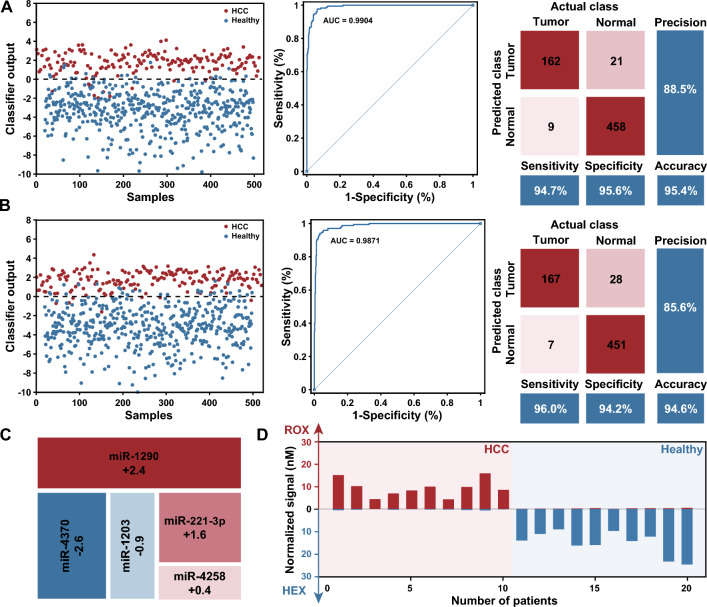
Profiling of the miRNA expression levels for the diagnosis of HCC. **A** In silico training of a minimal linear classifier to discriminate HCC from healthy individuals. Performance of the classifier with data from the training dataset, 94.7% and 95.6% of HCC from healthy individuals were classified correctly, with an AUC of 0.9904. **B** Performance of the classifier with data from the validation dataset, 96.0% and 94.2% of HCC from healthy individuals were diagnosed correctly, with an AUC of 0.9871. **C** Minimal linear classifier that included five miRNAs with weights ranging from  − 2.6 to 2.4. **D** Results of DNA-based molecular classifier for 10 cases each of HCC and healthy individuals use synthetic miRNAs

### Validation of the HCC diagnosis using synthetic miRNAs

To develop an effective classifier model for the in silico diagnosis of HCC, publicly available serum miRNA expression data corresponding to 345 HCC patients and 958 healthy individuals from GEO were used for classifier construction (details of the results are provided in Additional file 1: Text S4). First, differential expression analysis was used to identify miRNAs that were differentially expressed between the cancer and healthy groups. A total of 67 up-regulated and 174 down-regulated miRNA candidates exhibited expression level disparities that surpassed a fourfold magnitude. Then, a random-forest based algorithm was applied to assess the relevance of each signature by ranking them based on their predictive importance, and miRNAs were ranked by Mean Decrease Accuracy and Mean Decrease Gini. We subsequently designed a comprehensive SVM classifier consisting of 1 to 10 prominently ranked miRNAs in the training set, and selected a minimal set of miRNAs while maintaining classifier accuracy. It should be noted that the weights for each miRNA remained at one decimal place. In addition, the misclassification penalty for HCC samples was set to twice as high as that for healthy individuals, because an early diagnosis of HCC is crucial for improving its prognosis. Finally, we selected a classifier including five miRNAs with weights ranging from − 2.6 to 2.4 (Fig. 4C). The classifier discriminates between HCC and healthy samples with an area under the curve (AUC) of 0.9904 in the training dataset (171 HCC and 479 healthy samples) (Fig. 4A). The classifier model was further validated using an additional 174 HCC and 479 healthy samples, resulting in an AUC of 0.9871 (Fig. 4B). The classifier demonstrated excellent specificity and sensitivity, and allowed the implementation at the molecular level.

Next, we implemented the classifier by designing transformational and computational DNA circuits for miRNA inputs, and using synthetic miRNAs to evaluate the performance of well-designed molecular classifier. Ten patients each of HCC and healthy individuals correctly classified by the classifier in silico were selected and replicated in vitro. After transformation and DNA computation as illustrated before, fluorescence signals in the HEX and ROX channels were measured for each sample. The results showed that the expected signal was observed in the intended channel, while the signal remained near the background in the other channel (Fig. 4D). Moreover, we observed a robust correlation between the normalized signal intensity and the corresponding classifier output estimated in silico for each sample, indicating that our molecular classifier reproduced the SVM model (Additional file 1: Fig. S17).

**Fig. 5:**
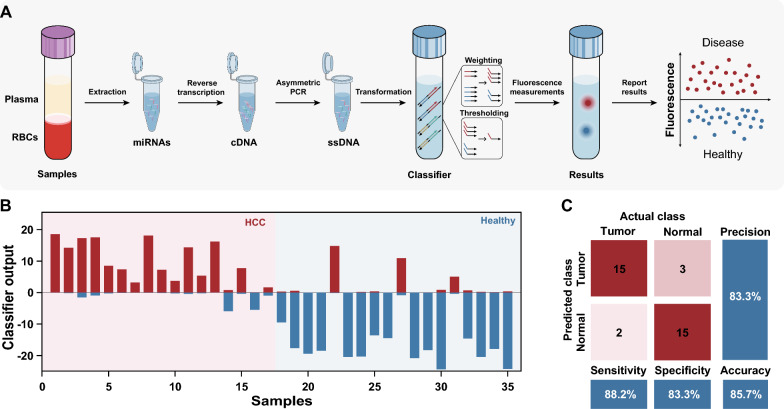
Profiling clinical samples by molecular classifier. **A** General workflow for molecular classifier to profile HCC clinical samples. **B** Profiling results for 17 patients with HCC and 18 healthy individuals. **C** Performance of the molecular classifier to discriminate HCC from healthy individuals

### Profiling clinical samples by molecular classifier

Finally, we verified the effectiveness of the molecular classifier for profiling HCC clinical samples. A general workflow is shown in Fig. 5A, miRNAs were first extracted from the plasma of each sample by a commercial kit, and reverse transcription and asymmetric PCR were subsequently performed to generate ssDNA, which was further transformed to corresponding inputs and processed by an established molecular classifier. The discrimination between HCC patients and healthy individuals was accomplished by monitoring the fluorescence signals in the HEX and ROX channels. The entire procedure takes approximately 3–4 h to complete.

The profiling results for 17 patients with HCC and 18 healthy individuals are shown in Fig. 5B and Additional file 1: Fig. S18. 15 out of 17 patients with HCC were diagnosed correctly with a sensitivity of 88.2%, 3 of 18 healthy individuals were misdiagnosed with a specificity of 83.3%. The total accuracy of the classifier for HCC diagnosis in clinical samples was 85.7% (Fig. 5C). Indeed, the results demonstrate the tremendous potential of our method in clinical diagnosis.

In our approach, some improvements were developed to drive the adoption and implementation of molecular classifiers in clinical settings. First, asymmetric PCR followed by subsequent associative strand displacement was used to modularly decouple sequence constraints between RNAs and molecular classifiers, which enables the extensive use of molecular classifiers across various gene expression signatures. Furthermore, for RNA transcripts with intricate secondary structures, associative strand displacement can be accomplished by hybridizing helper strands adjacent to the targeted region on ssDNA [21]. Second, the competitive inhibition system enables precise weight assignment for different inputs, which better aligns with the continuous optimization process of machine learning and accurately captures the importance of RNAs. The molecular implementation will accelerate more application of machine learning models in personalized diagnostics. Finally, by adjusting the sequence of DNA domains to control the reaction rate and orthogonality for different inputs, the molecular classifier could in principle be scaled to dozens or even hundreds of gene expression signatures. Overall, with the decreasing cost of synthetic DNA and advancements in microfluidics technology, an effective diagnostic model and a powerful DNA-based molecular classifier can be integrated into a completely automated classification workflow, this integration may facilitate a standardized testing process in low-resource settings.

Nevertheless, more efforts are needed to propel molecular classifiers from research settings to routine clinical practice. For instance, the introduction of an automated system could shorten the turnaround time of experiments and minimize human errors [38]. More testing of the classifier in large and diverse patient populations should be performed to ensure its robustness and generalizability. Optimization of the classifier’s parameters and algorithms may also be performed to enhance its predictive power. We believe future highly integrated DNA-based molecular classifiers may offer universality and scalability by allowing for the encoding of higher valence numbers and hence the detection of larger panels of biomarkers.

## Conclusions

In summary, we developed a systematic workflow, based on DNA molecular classifiers, to translate gene expression signatures into clinical interpretations. This method accurately classified HCC patients and healthy individuals with five miRNAs from blood samples. Given the exciting results, we envision that DNA-based molecular classifiers will advance clinical studies that explore the correlation between gene expression patterns and disease diagnostics, monitoring, and prognosis. Moreover, this progress will support advocacy for personalized medicine.

### Supplementary Information

**Additional file 1: Text S1.** The Concentration Relationship between cDNA and the Generated ssDNA in Asymmetric PCR. **Text S2.** Reaction Model Analysis: Irreversible Competitive Inhibition. **Text S3.** Reaction Model Analysis: Molecular implementation of Irreversible Competitive Inhibition. **Text S4.** Classifier model for diagnosis of liver cancer in silico. **Fig. S1.** Fitting of rate constants. **Fig. S2.** Kinetics for different concentration of Input. **Fig. S3.** Calibrated model for different concentration of Input. **Fig. S4.** Simulation of the irreversible competitive inhibition system. **Fig. S5.** Generality of the weighting. **Fig. S6.** miRNAs expression fold changes between the HCC and healthy samples. **Fig. S7.** miRNAs ranked by random-forest based algorithm. **Fig. S8.** Distributions of top 10 ranked miRNAs between the HCC and healthy samples. **Fig. S9.** The diagnostic power of single miRNA. **Fig. S10.** The diagnostic power of multiple miRNA. **Fig. S11.** PCR efficiency calculation. **Fig. S12.** Optimizing the hybridization length between ssDNA and splitting modules. **Fig. S13.** Optimizing length of junction between two modules. **Fig. S14.** Optimizing split position distance from toehold to minimize leakage in the process of conversion. **Fig. S15.** Standard curve of each reporter complex. **Fig. S16.** Optimizing the length of toehold on the annihilator. **Fig. S17.** Performance of the optimized annihilator. **Fig. S18.** Correlation between the normalized signal intensity and the corresponding classifier output. **Fig. S19.** Profiling results for 17 patients with HCC and 18 healthy individuals. **Fig. S20.** Strand name for the sequences. **Table S1.** Sequences of synthetic miRNA and corresponding primer. **Table S2.** Sequences of strands in programmable transformation strategy. **Table S3.** Sequences of strands in classification model.

## Data Availability

The characterization data and experimental protocols for this work are available within this manuscript and its associated Additional Information, or from the corresponding author upon request.
