# Supplementary material for: DNA-based molecular classifiers for the profiling of gene expression signatures
Source: J Nanobiotechnology. 2024 Apr 17;22:189. doi: 10.1186/s12951-024-02445-0 (PMC11025223; doi:10.1186/s12951-024-02445-0)
Supplement: Supplementary file 1 — Additional file 1: Text S1. The Concentration Relationship between cDNA and the Generated ssDNA in Asymmetric PCR. Text S2. Reaction Model Analysis: Irreversible Competitive Inhibition. Text S3. Reaction Model Analysis: Molecular implementation of Irreversible Competitive Inhibition. Text S4. Classifier model for diagnosis of liver cancer in silico. Fig. S1. Fitting of rate constants. Fig. S2. Kinetics for different concentration of Input. Fig. S3. Calibrated model for different concentration of Input. Fig. S4. Simulation of the irreversible competitive inhibition system. Fig. S5. Generality of the weighting. Fig. S6. miRNAs expression fold changes between the HCC and healthy samples. Fig. S7. miRNAs ranked by random-forest based algorithm. Fig. S8. Distributions of top 10 ranked miRNAs between the HCC and healthy samples. Fig. S9. The diagnostic power of single miRNA. Fig. S10. The diagnostic power of multiple miRNA. Fig. S11. PCR efficiency calculation. Fig. S12. Optimizing the hybridization length between ssDNA and splitting modules. Fig. S13. Optimizing length of junction between two modules. Fig. S14. Optimizing split position distance from toehold to minimize leakage in the process of conversion. Fig. S15. Standard curve of each reporter complex. Fig. S16. Optimizing the length of toehold on the annihilator. Fig. S17. Performance of the optimized annihilator. Fig. S18. Correlation between the normalized signal intensity and the corresponding classifier output. Fig. S19. Profiling results for 17 patients with HCC and 18 healthy individuals. Fig. S20. Strand name for the sequences. Table S1. Sequences of synthetic miRNA and corresponding primer. Table S2. Sequences of strands in programmable transformation strategy. Table S3. Sequences of strands in classification model. [file 12951_2024_2445_MOESM1_ESM.pdf]

DNA-based molecular classifiers for the profiling of gene expression signatures

Li Zhang[1,2†], Qian Liu[3†], Yongcan Guo[4], Luyao Tian[1], Kena Chen[1], Dan Bai[1], Hongyan Yu[1], Xiaole Han[1], Tong Feng[1], Shixiong Deng[2*], Guoming Xie[1*]

[1]Key Laboratory of Laboratory Medical Diagnostics, Ministry of Education, Department of Laboratory Medicine, Chongqing Medical University, Chongqing, 400016, China.
[2]Department of Forensic Medicine, Chongqing Medical University, Chongqing, 400016, China.
[3]Nuclear Medicine Department, The Second Affiliated Hospital of Chongqing Medical University, Chongqing, 400010, China.
[4]Clinical Laboratory, Traditional Chinese Medicine Hospital Affiliated to Southwest Medical University, Luzhou, 646000, China.

*Corresponding author(s). E-mail(s): dengshixiong@cqmu.edu.cn; guomingxie@cqmu.edu.cn;
†These authors contributed equally to this work.

Contents

1 Supplementary Text **1**
1.1 Text S1. The Concentration Relationship between cDNA and the Generated ssDNA in Asymmetric PCR 1
1.2 Text S2. Reaction Model Analysis: Irreversible Competitive Inhibition 1
1.3 Text S3. Reaction Model Analysis: Molecular implementation of Irreversible Competitive Inhibition 2
1.4 Text S4. classifier model for diagnosis of liver cancer in silico 10

Supplementary Figures

Fig. 1 Fitting of rate constants 4
Fig. 2 Kinetics for different concentration of *Input* 5
Fig. 3 Calibrated model for different concentration of *Input* 6
Fig. 4 Simulation of the irreversible competitive inhibition system 7
Fig. 5 Generality of the weighting 9
Fig. 6 miRNAs expression fold changes between the HCC and healthy samples 10
Fig. 7 miRNAs ranked by random-forest based algorithm 11
Fig. 8 Distributions of top 10 ranked miRNAs between the HCC and healthy samples 12
Fig. 9 The diagnostic power of single miRNA 13
Fig. 10 The diagnostic power of multiple miRNA 14
Fig. 11 PCR efficiency calculation 15
Fig. 12 Optimizing the hybridization length between ssDNA and splitting modules 16
Fig. 13 Optimizing length of junction between two modules 17
Fig. 14 Optimizing split position distance from toehold to minimize leakage in the process of conversion 18
Fig. 15 Standard curve of each reporter complex 19
Fig. 16 Optimizing the length of toehold on the annihilator 20
Fig. 17 Performance of the optimized annihilator 21
Fig. 18 Correlation between the normalized signal intensity and the corresponding classifier output 22
Fig. 19 Profiling results for 17 patients with HCC and 18 healthy individuals 23
Fig. 20 Strand name for the sequences 24

Supplementary Tables

Table 1 Sequences of synthetic miRNA and corresponding primer 25
Table 2 Sequences of strands in programmable transformation strategy . 26
Table 3 Sequences of strands in classification model 29

1 Supplementary Text

1.1 Text S1. The Concentration Relationship between cDNA and the Generated ssDNA in Asymmetric PCR

In asymmetric PCR, depletion of the limiting primer during the exponential amplification results in the linear synthesis of the single strand extended from the excess primer. Generated ssDNA follows the equation:

$$C_{sn} = C_L \times (n - log_{(1+E)} \frac{C_L}{C_0}) \tag{1}$$

where C_{sn} is the concentrations of generated ssDNA after n cycles, C_L and C_0 are the concentrations of limiting primer and initial cDNA, and E is the efficiency of the PCR. For different cDNA (C_{01}, C_{02}), the ratio of generated ssDNA (C_{ss1}, C_{ss2}) can be calculated as:

$$\frac{C_{ss1}}{C_{ss2}} = \frac{C_L \times (n - log_{(1+E)} \frac{C_L}{C_{01}})}{C_L \times (n - log_{(1+E)} \frac{C_L}{C_{02}})} \tag{2}$$

$$= \frac{n - log_{(1+E)} C_L + log_{(1+E)} C_{01}}{n - log_{(1+E)} C_L + log_{(1+E)} C_{02}} \tag{3}$$

It is clear that, when $n - log_{(1+E)} C_L = 0$, that's after $n = log_{(1+E)} C_L$ cycles, equation (2) can be rewritten as:

$$\frac{C_{ss1}}{C_{ss2}} = \frac{log_{(1+E)} C_{01}}{log_{(1+E)} C_{02}} \tag{4}$$

Equation (4) confirms that, after $n = log_{(1+E)} C_L$ cycles, the ratio of generated ssDNA is consistent with those of logarithmic initial concentrations of the cDNA. Therefore, we employed asymmetric PCR to achieve nearly linear amplification of RNAs, relative to their logarithmic initial concentrations.

1.2 Text S2. Reaction Model Analysis: Irreversible Competitive Inhibition

To implement arbitrary weight assignment at the molecular level, we designed an DNA catalytic system with inhibitor. Similar to the irreversible competitive inhibition model of enzymatic reactions:

$$Input + Amplifier \xrightarrow{k} Input + Output \tag{5}$$

$$Input + Inhibitor \xrightarrow{k} Waste \tag{6}$$

In the reaction (5), the $Input$ acts as a catalyst to transform substrate $Amplifier$ into a product $Output$ with a rate constant of k. At the same time, the $Input$ can also participate in $Inhibitor$ to generate inactive products $Waste$ with a same rate

constant.The corresponding ordinary differential equations are:

$$\frac{d[Input]}{dt} = -k[Input][Inhibitor] \tag{7}$$

$$\frac{d[Output]}{dt} = k[Input][Amplifier] \tag{8}$$

$$\frac{d[Amplifier]}{dt} = -k[Input][Amplifier] \tag{9}$$

$$\frac{d[Inhibitor]}{dt} = -k[Input][Inhibitor] \tag{10}$$

The differential equations resulting from this model can be integrated with initial conditions $[Input](0) = [Input]_0$, $[Output](0) = 0$, $[Amplifier](0) = [Amplifier]_0$,and $[Inhibitor](0) = [Inhibitor]_0$. Solving for the product [Output](t):

$$[Output](t) = [Amplifier]_0 - [Amplifier]_0(\frac{1-\alpha}{1-\alpha e^{k([Input]_0-[Inhibitor]_0)}}) \tag{11}$$

where α is the ratio of initial concentration of *Input* and *Inhibitor*: $\alpha = [Input]_0/[Inhibitor]_0$. The final concentration of generated *Output* can be computed by taking the limit $t \to \infty$:

$$\lim_{t\to\infty}[Output](t) = \begin{cases} [Amplifier]_0, & [Input]_0 > [Inhibitor]_0 \\ [Amplifier]_0 \times \alpha, & [Input]_0 \leq [Inhibitor]_0 \end{cases} \tag{12}$$

Rationally, if the initial *Input* concentration is greater than the *Inhibitor*, excess *Inputs* will eventually catalyze all the *Amplifier* into *Output*. The other situation can be more interesting, when $[Input]_0 \leq [Inhibitor]_0$, final concentration of *Output* is linear in the initial concentration of *Input*:

$$[Output]_\infty = [Input]_0 \frac{[Amplifier]_0}{[Inhibitor]_0} \tag{13}$$

Equetion (13) implies that we can implement any weight to *Input* by adjusting the initial relative concentration of *Amplifier* and *Inhibitor*:

$$[Output]_\infty = w \times [Input]_0 \tag{14}$$

1.3 Text S3. Reaction Model Analysis: Molecular implementation of Irreversible Competitive Inhibition

Ordinary Differential Equations

Herein, we designed an DNA based reaction network to implemnt irreversible competitive inhibition. As shown in Figure 2A, an entropy driven-catalytic system acts as

the the amplifier, and a similar cascade reaction as corresponding inhibitor maintain a consistent reaction rate to input. All relevant reactions in the system are:

$$Input + Amplifier \underset{k_{1r}}{\overset{k_{1f}}{\rightleftharpoons}} AI + W \tag{15}$$

$$Input + Inhibitor \underset{k_{1r}}{\overset{k_{1f}}{\rightleftharpoons}} InI + W \tag{16}$$

$$AI + AF \xrightarrow{k_2} Input + AW + Output \tag{17}$$

$$InI + IF \xrightarrow{k_2} SW1 + SW2 \tag{18}$$

The corresponding ordinary differential equations (ODEs) are:

$$\frac{d[Input]}{dt} = -k_{1f}[Input][Amplifier] + k_{1r}[AI][W] - k_{1f}[Input][Inhibitor] + k_{1r}[InI][W] + k_2[AI][AF] \tag{19}$$

$$\frac{d[Amplifier]}{dt} = -k_{1f}[Input][Amplifier] + k_{1r}[AI][W] \tag{20}$$

$$\frac{d[Inhibitor]}{dt} = -k_{1f}[Input][Inhibitor] + k_{1r}[InI][W] \tag{21}$$

$$\frac{d[AI]}{dt} = k_{1f}[Input][Amplifier] - k_{1r}[AI][W] - k_2[AI][AF] \tag{22}$$

$$\frac{d[W]}{dt} = k_{1f}[Input][Amplifier] - k_{1r}[AI][W] + k_{1f}[Input][Inhibitor] - k_{1r}[InI][W] \tag{23}$$

$$\frac{d[SI]}{dt} = k_{1f}[Input][Inhibitor] - k_{1r}[InI][W] - k_2[SI][SF] \tag{24}$$

$$\frac{d[AF]}{dt} = -k_2[AI][AF] \tag{25}$$

$$\frac{d[SF]}{dt} = -k_2[SI][SF] \tag{26}$$

$$\frac{d[AW]}{dt} = k_2[AI][AF] \tag{27}$$

$$\frac{d[Output]}{dt} = k_2[AI][AF] \tag{28}$$

$$\frac{d[SW1]}{dt} = k_2[SI][SF] \tag{29}$$

$$\frac{d[SW2]}{dt} = k_2[SI][SF] \tag{30}$$

wherein k_{1f} and k_{1r} represent the forward and the reverse rate constant for the reactions between $Input$ and $Amplifier$ or $Inhibitor$. k_2 represents the rate constants for reactions between intermediates and fuels.

Fitting of rate constants

We designed a secondary reporter systems for calculating these rate constants. Reporter had a 6-nt toehold domain as a single-stranded 5' overhang and a 16-nt displacement domain, which complementary to *Output* strand. Initially, the requisite toehold domain of *Output* is sequestered within the *Amplifier* and the displacement domain exists as a single-stranded 5' overhang. Once the *Output* was released, free *Outputs* activate the reporter immediately with a rate constant of k_{rep}.

$$Output + Reporter \xrightarrow{k_{rep}} Fluorescence + Waste \tag{31}$$

Additionally, to minimize the leakage arise from spontaneous fraying that reveals the toehold in *Amplifier*, toehold of reporter was more effectively sequestered within the *Amplifier*, starting 6 nt from the end of the duplex. Fluorescence data were zeroed by subtracting from all fluorescence counts the fluorescence of the last data point collected before addition of reactant and subsequently converted to normalized signal.

As described in detail below, we processed and fitted the data obtained from the reporter system to estimate the rate constants associated with each individual reaction contributing to the observed behavior. An objective function was defined to calculate the goodness of fit for a given set of rate constants. The function computes the difference between the normalized signal and the numerical solution obtained by solving the ODEs using the MATLAB function $ode15s$, from t_0 to t_{end}. An excessively restrictive additional penalty is imposed in the event that any of the parameters are negative. The objective function is minimized with MATLAB function $fminsearch$, initial values of k_{1f}, k_{1r}, k_2 and k_{rep} were set to 1×10^5.

Fittd results were shown in Figure S1, the report rate constant k_{rep} is 7.0×10^5 $M^{-1}s^{-1}$, which consistent with previous studies. On this basis, k_2 was fitted to 3.7×10^5 $M^{-1}s^{-1}$. The values of the forward and reverse rate constants were calculated as 4.3×10^5 $M^{-1}s^{-1}$ and 1.1×10^3 $M^{-1}s^{-1}$ respectively, which were subjected to De Donder equality:

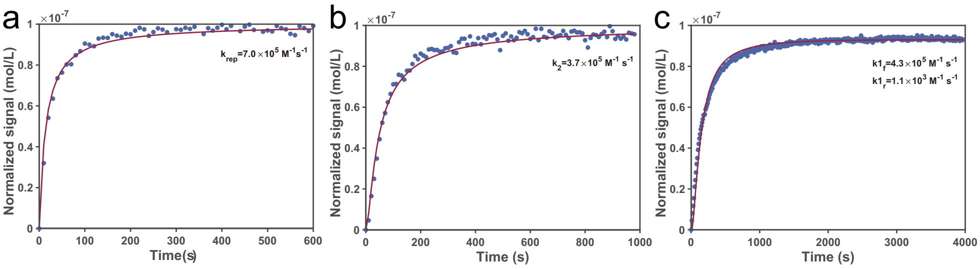

Figure. S 1: Fitting of rate constants. (a) Zeroed fluorescence data for reporting reaction (blue points). Fitted k_{rep}(corresponding to red curve). (b) Fitted k_2. (c) Fitted k_{1f} and k_{2f}.

$$\frac{k1_f}{k1_r} = K_{\text{eq}} = e^{\Delta G^{\text{o}}_{\text{rxn}}/R\tau} \tag{32}$$

where $\Delta G^{\text{o}}_{\text{rxn}}$ is the standard free energy of the relevant reaction, R is gas constant, τ is temperature.

Calibration of model

We simulated the catalytic system using the measured rate constants and experimentally investigate the kinetics for different concentration of *Input*. As shown in Figure S2, in an ideal catalytic system, the catalyst facilitates the conversion of the substrate into the product until all the substrates are completely consumed. However, we have observed that as the concentration of the catalyst decreases, the maximum conversion yield of the substrate gradually diminishes. It is looks like the deactivation of the catalyst caused by side reactions, which are widely encountered in enzymatic reactions. Side reactions in DNA based reaction mainly caused by DNA strand impurities and

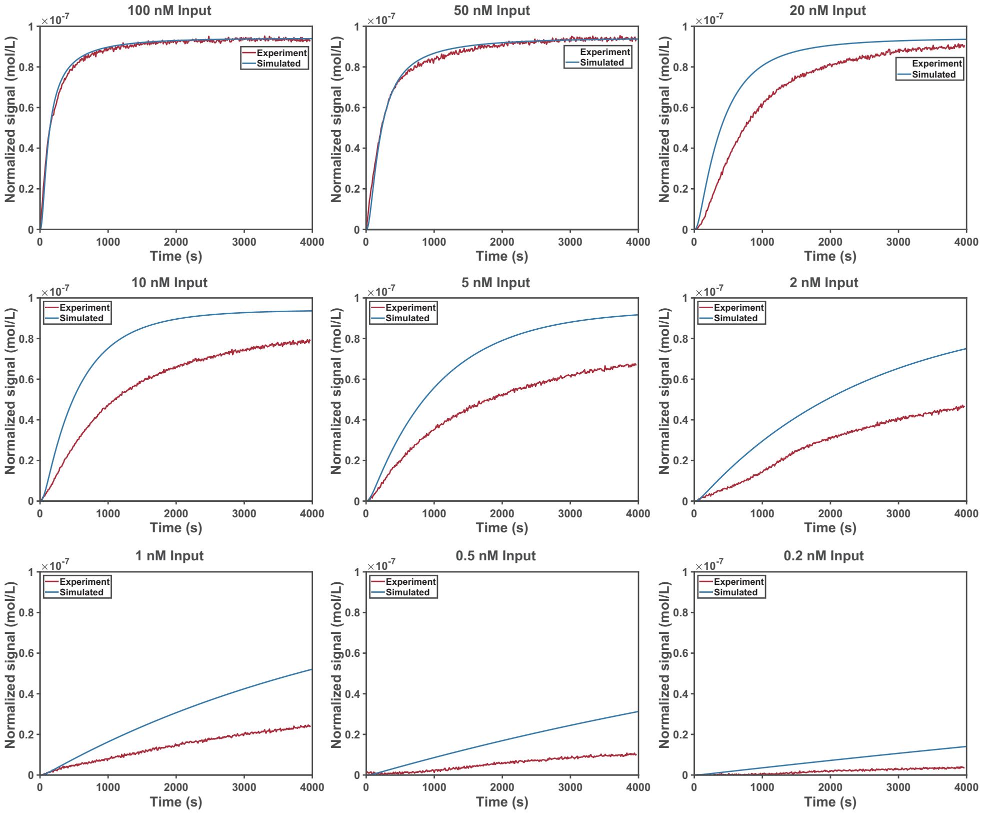

Figure. S 2: Kinetics for different concentration of *Input*.

misfolding complexes. For example, error nucleotides in fuel strand impede the release

of catalyst $Input$ from intermediate AI, resulting in deactivation of the $Input$. Therefore, we modified the model to calibrate the influence of side reactions. We assumed that a certain proportion of fuel strand AF are 'sicked', referred to as SF. SF can react with AI to yield a new product SI, which is incapable of releasing $Input$. The reaction was described as follow:

$$AI + SF \xrightarrow{k_3} SI + Output \tag{33}$$

Calibrated model was shown in Figure S3, the proportion of SF is estimated to be 1% for HPLC-purified strands. Calibrated model quantitatively predicts the kinetics for different concentration of $Input$.

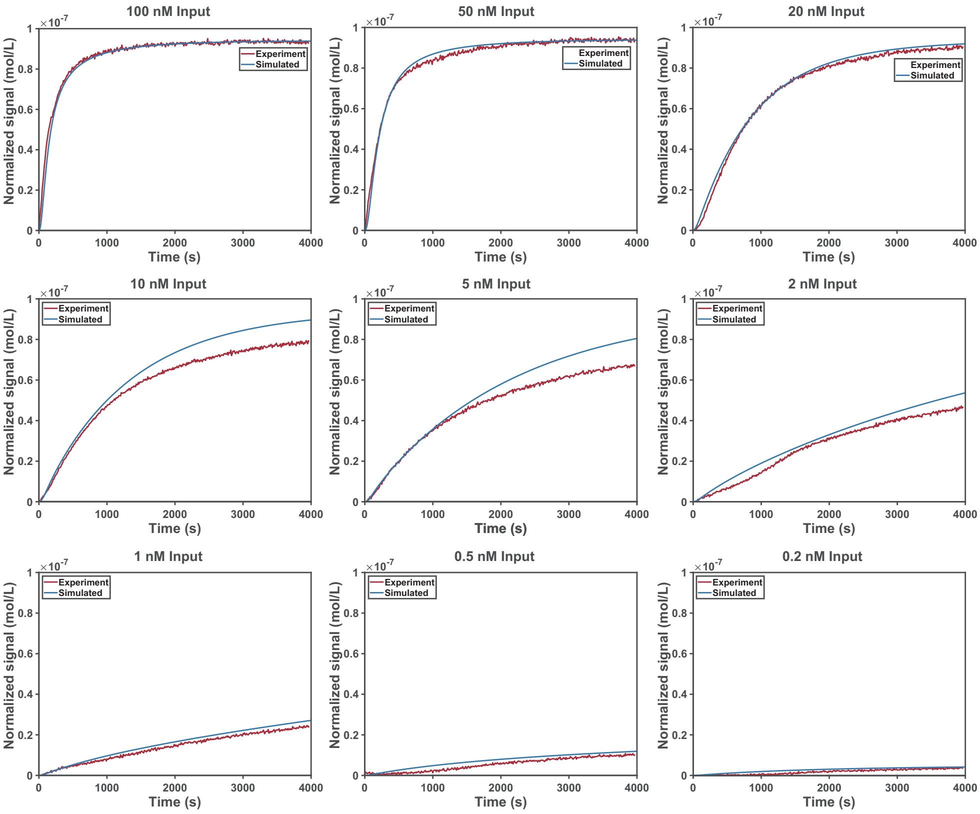

Figure. S 3: Calibrated model for different concentration of $Input$.

Irreversible Competitive Inhibition Enable Arbitrary Weighting

In the irreversible competitive inhibition system, amplification and inhibition reactions were initiated with a reversible toehold exchange to ensure same rate constant. Subsequently, amplification intermediate AI was incorporated by AF and release $Output$

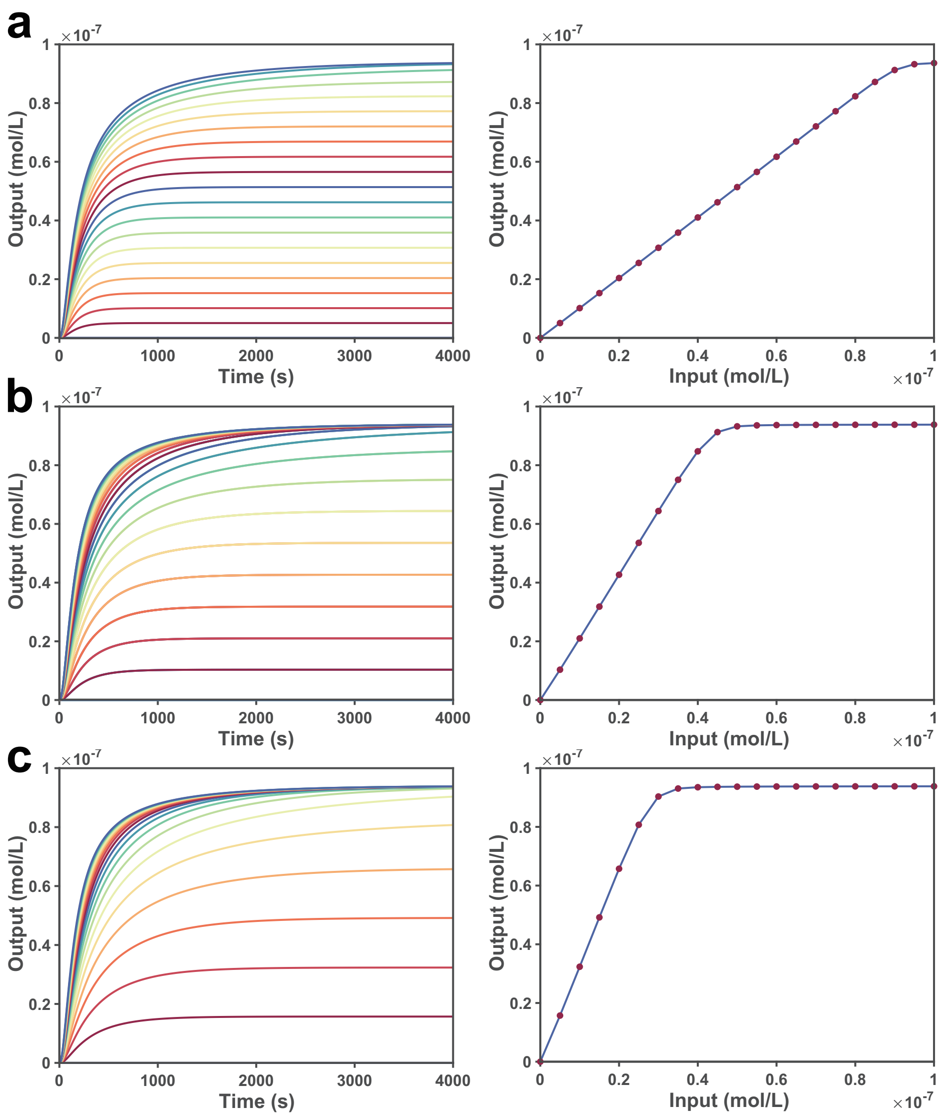

Figure. S 4: Weights w = (a) 1, (b) 2, (c) 3 for $Input$ from 0 nM to 100 nM. $Amplifier$ = 100 nM, $Inhibitor$ = 89 nM, 42 nM, 27 nM.

and $Input$ at the same time. On the contrary, inhibition intermediate InI was inactivated by IF, resulting in the absence of any output. This mechanism exhibits similarity to the side reactions encountered in enzymatic reactions.

Based on the calibrated model, we simulated the irreversible competitive inhibition system to predict its capability in enabling arbitrary weighting. Firstly, weights $w = 1$, 2, 3 for $Input$ from 0 nM to 100 nM were simulated in Figure S4. The results indicate

that, when $Input$ concentration is lower than that of the $Inhibitor$:

$$[Output] = w \times [Input] \tag{34}$$

Once the $Input$ concentration surpasses that of the $Inhibitor$:

$$[Output] = [Amplifier] \tag{35}$$

Next, we simulated $w = 1$, 1.5, 2, 2.5, 3, 3.5, 4, 4.5, 5 for $Input$ concentration of 1 nM, 5 nM, 10 nM to validate the generality of the weighting. As shown in Figure S5, we found that as the input concentration increases, there is a noticeable deviation in the accuracy of the weighting. Moreover, the degree of deviation intensifies as the weights increase. We speculated that this deviation is caused by depletion of $Amplifier$ and $Inhibitor$. In subsequent experiments, we employed a weight range of 0 to 3 while ensuring that the concentration of the $Inhibitor$ remained significantly higher than that of the $Input$.

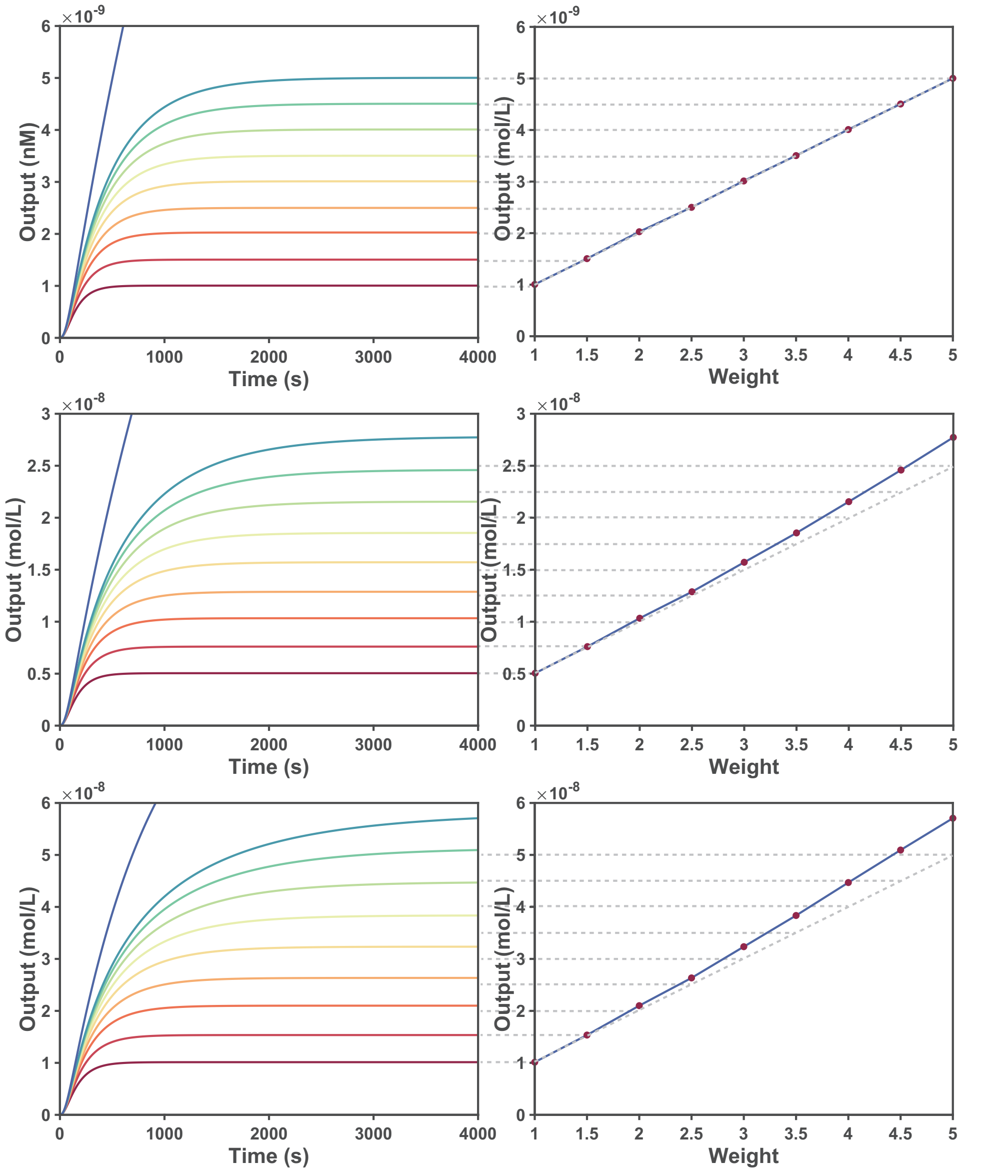

Figure. S 5: Weights $w = 1, 1.5, 2, 2.5, 3, 3.5, 4, 4.5, 5$ for $Input$ concentration of 1 nM, 5 nM, 10 nM. $Amplifier = 100$ nM.

1.4 Text S4. classifier model for diagnosis of liver cancer in silico

To build a molecular classifier, we simplified the classification problem by distinguishing only between hepatocellular carcinoma (HCC) and healthy individuals. A publicly available serum microRNA profiles data corresponding to 345 HCC and 958 healthy individuals were used for classifier training. Firstly, differential expression analysis was used to identify miRNAs that were differentially expressed between cancer and healthy groups. As shown in Figure S6, a total of 67 up-regulated and 174 down-regulated miRNA candidates revealed expression level disparities surpassing a fourfold magnitude.

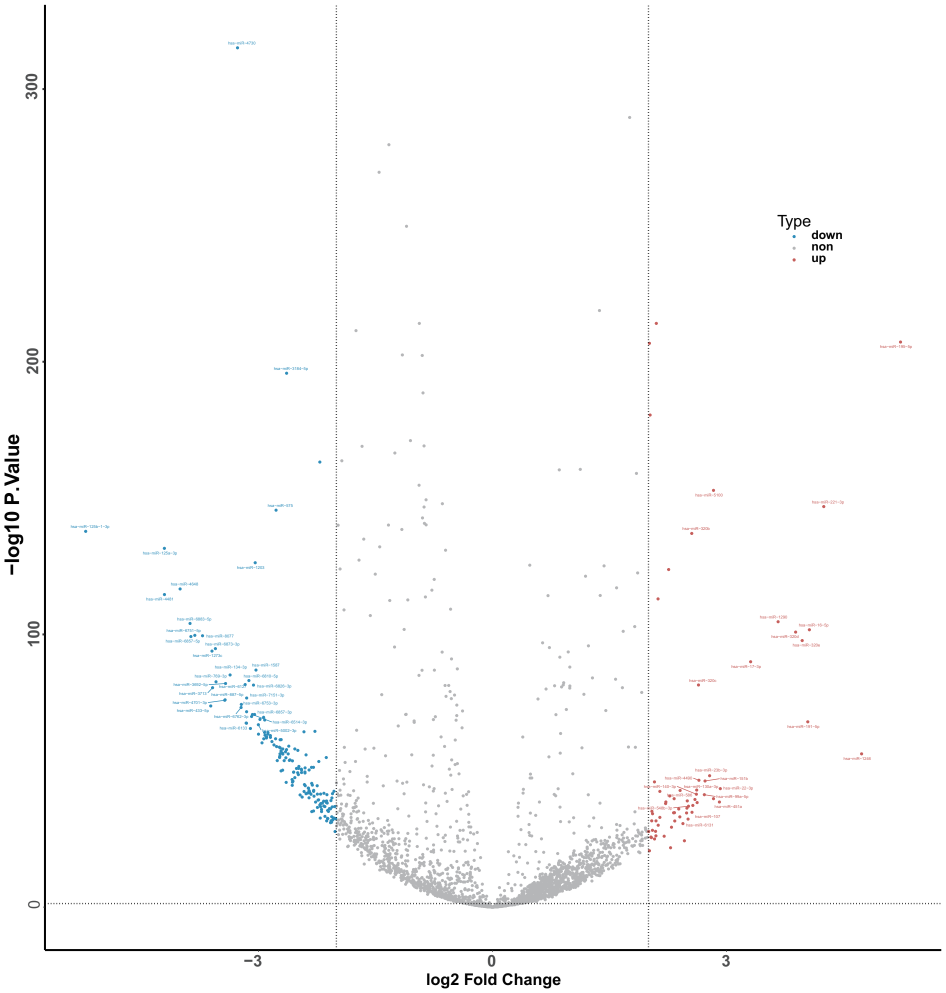

Figure. S 6: miRNAs expression fold changes between the HCC and healthy samples. Colored data are shown with a $|Fold\ change|>4$.

Then, a random-forest based algorithm was applied to assess the relevance of each signature by ranking them based on their predictive importance, and miRNAs ranked by Mean Decrease Accuracy (MDA) and Mean Decrease Gini (MDG). Figure S7 displays the top 20 ranked miRNAs in MDA and MDG. We selected 10 miRNAs that ranked highly in both rankings for classifier construction. Expression levels of 10 selected miRNAs between the HCC and healthy samples were shown in Figure S8.

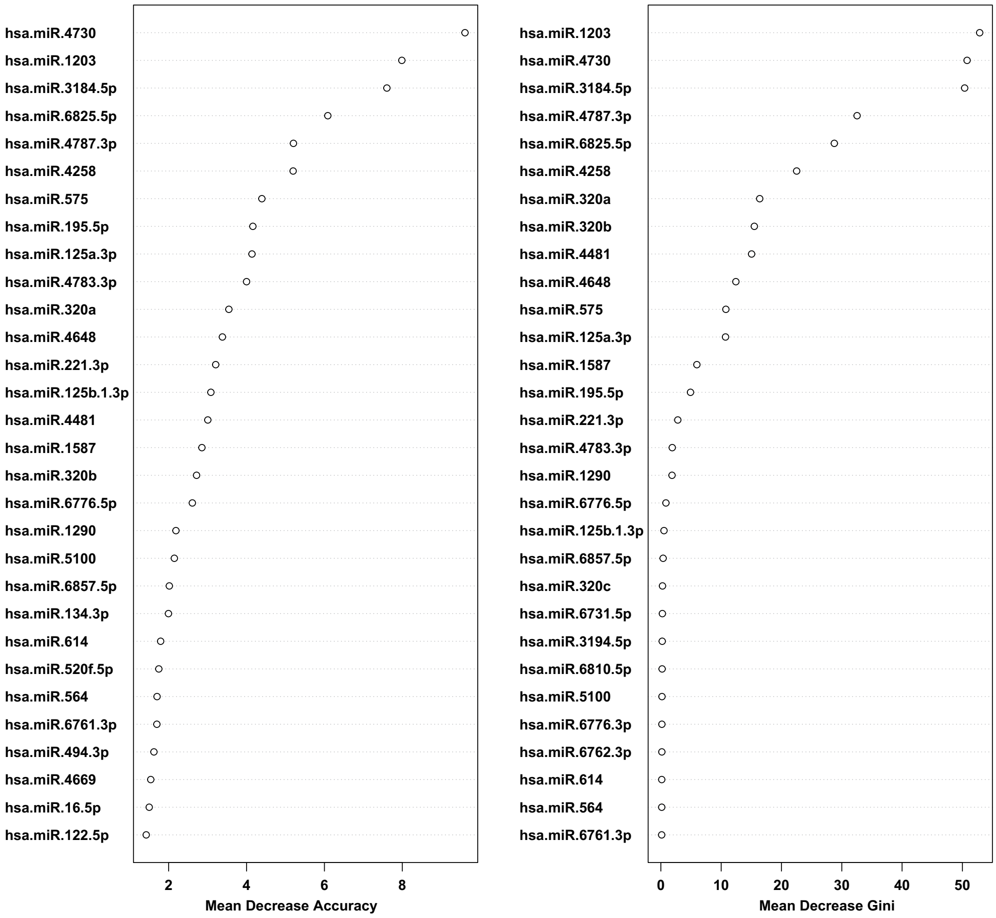

Figure. S 7: miRNAs ranked by Mean Decrease Accuracy and Mean Decrease Gini

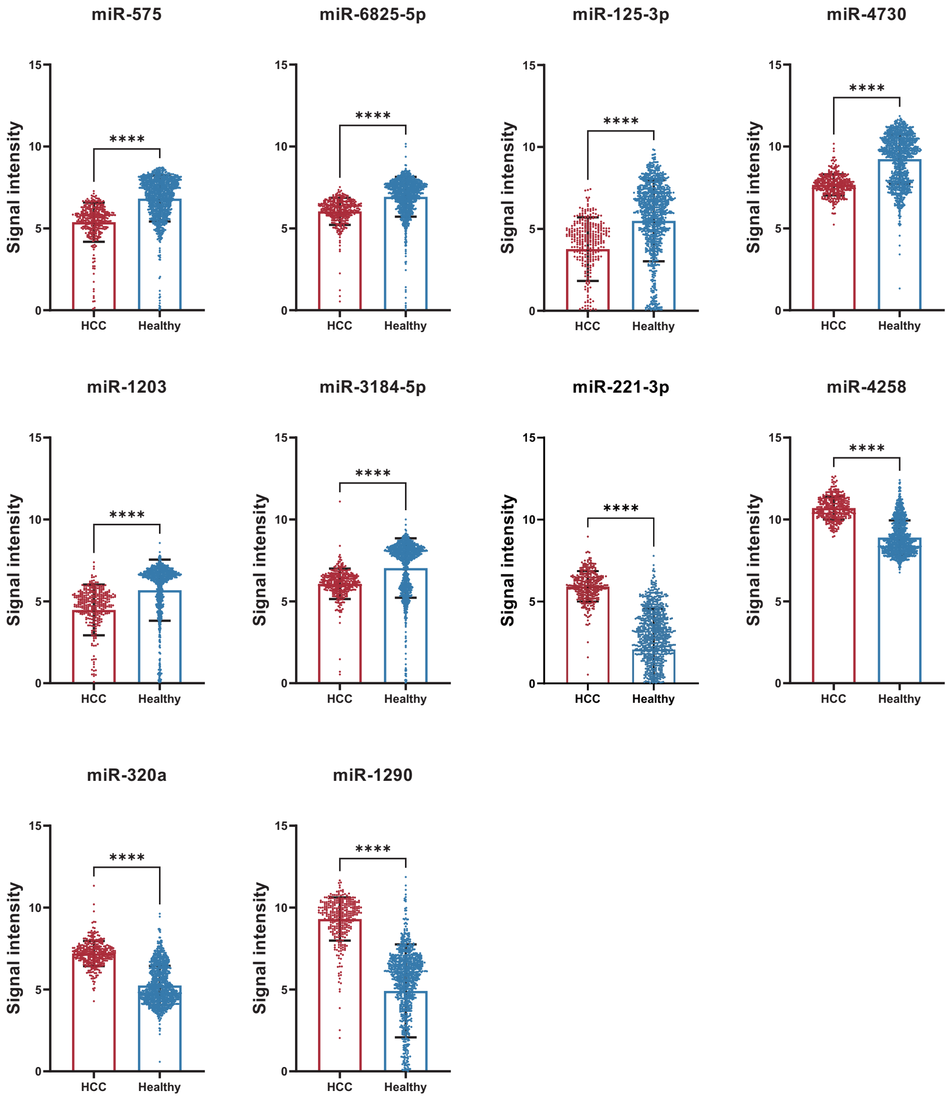

Figure. S 8: Distributions of top 10 ranked miRNAs between the HCC and healthy samples. Fold change, unpaired Student t test, **** P <0.00001.

These samples were randomly separated into the training dataset (171 HCC and 479 healthy samples) and validation dateset (174 HCC and 479 healthy samples). Support vector machine (SVM) was used to train the data. As illustrated in Figure S9, the diagnostic power of a single miRNA is unsatisfactory.

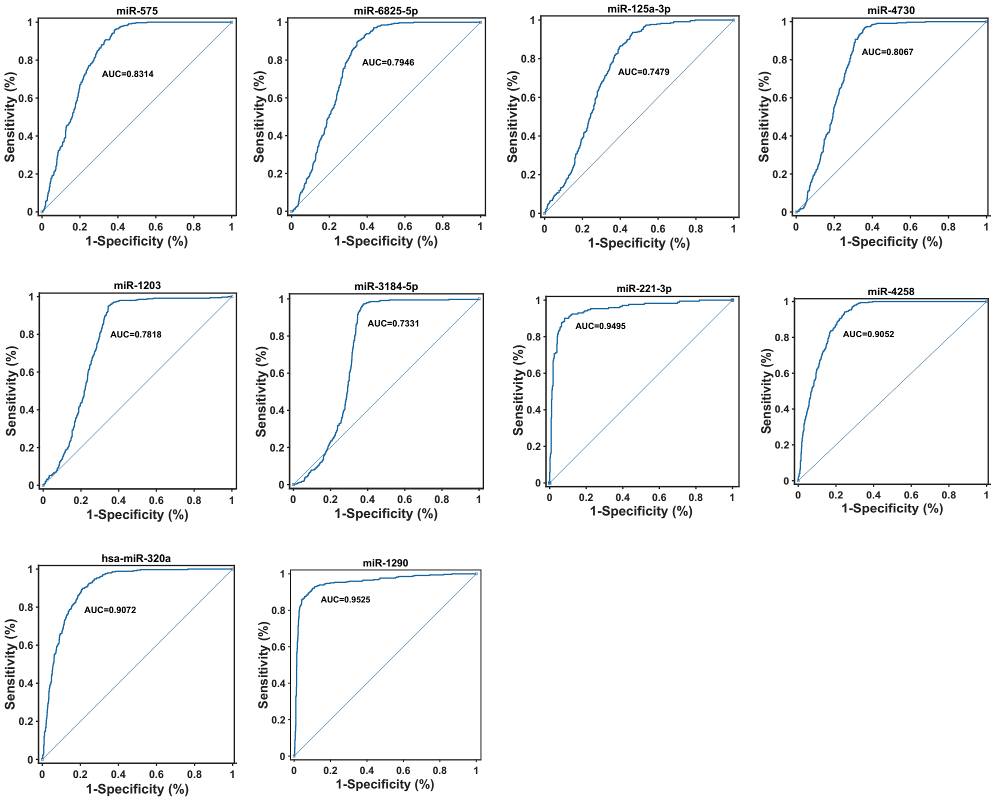

Figure. S 9: The diagnostic power of single miRNA

We subsequently trained an SVM classifier (with a linear kernel) consisting of 1 to 10 prominently ranked miRNAs. Some restrictions were incorporated into the algorithm: weights are artificially limited to values remain one decimal place between -3 to +3, this choice was made to ensure the accuracy; the misclassification penalty for HCC samples was set to be twice as high as that for healthy individuals, because an early diagnosis of HCC is crucial for improving its prognosis. Figure S10 shows the optimal diagnostic performance of the panel comprising 1 to 10 miRNAs. As the number of miRNAs increases from 1 to 5, the accuracy of the model significantly improved. Following the subsequent increase in the number of miRNAs, the accuracy of the model remains relatively stable. Finally, a classifier was identified utilizing a combination of five miRNAs (miR-221-3p, miR-1203, miR-1290, miR-4258, miR-4370) with weights ranging from -2.6 to 2.4. The selected classifier correctly labelled 94.7% and 95.6% of HCC and healthy samples in the training set and 96.0% of HCC and 94.2% of healthy samples in the validation set.

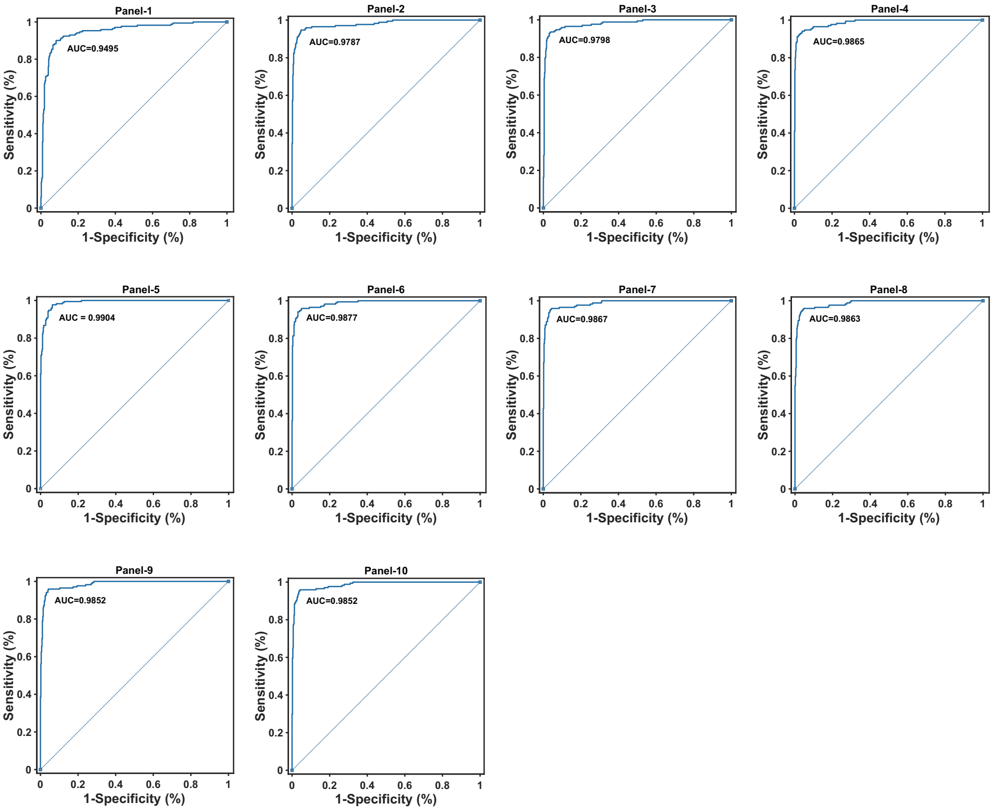

Figure. S 10: The diagnostic power of multiple miRNA

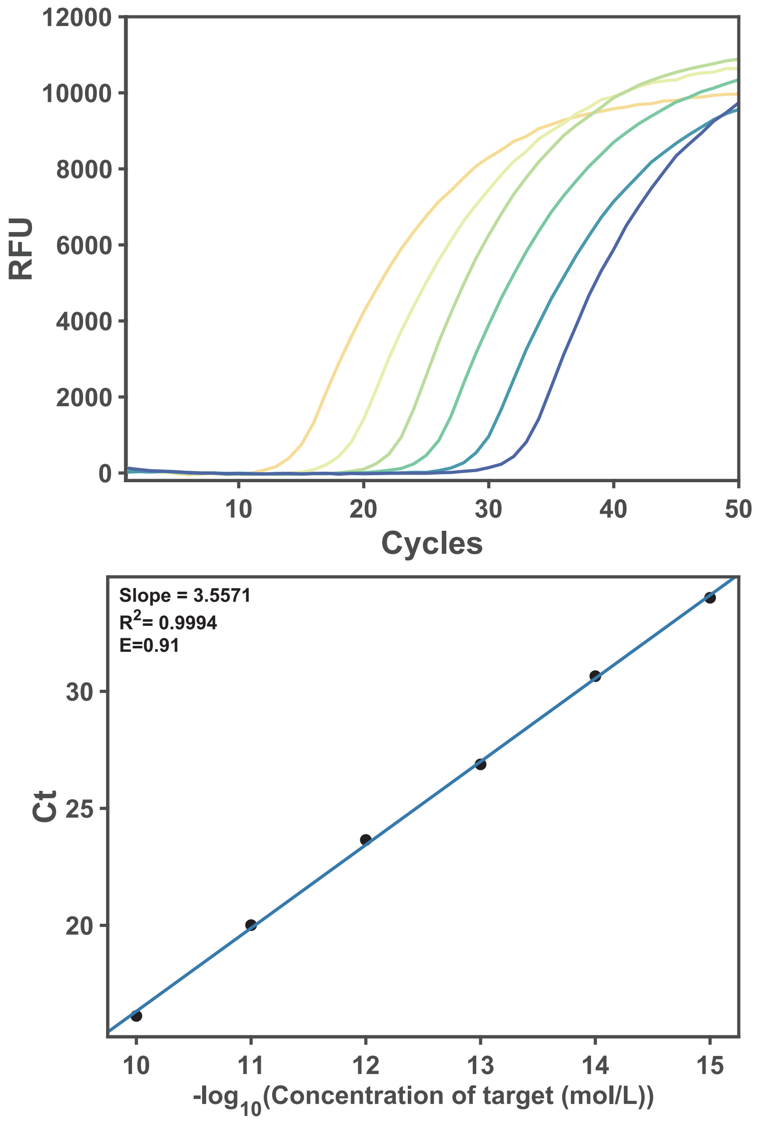

Figure. S 11: PCR efficiency calculation.

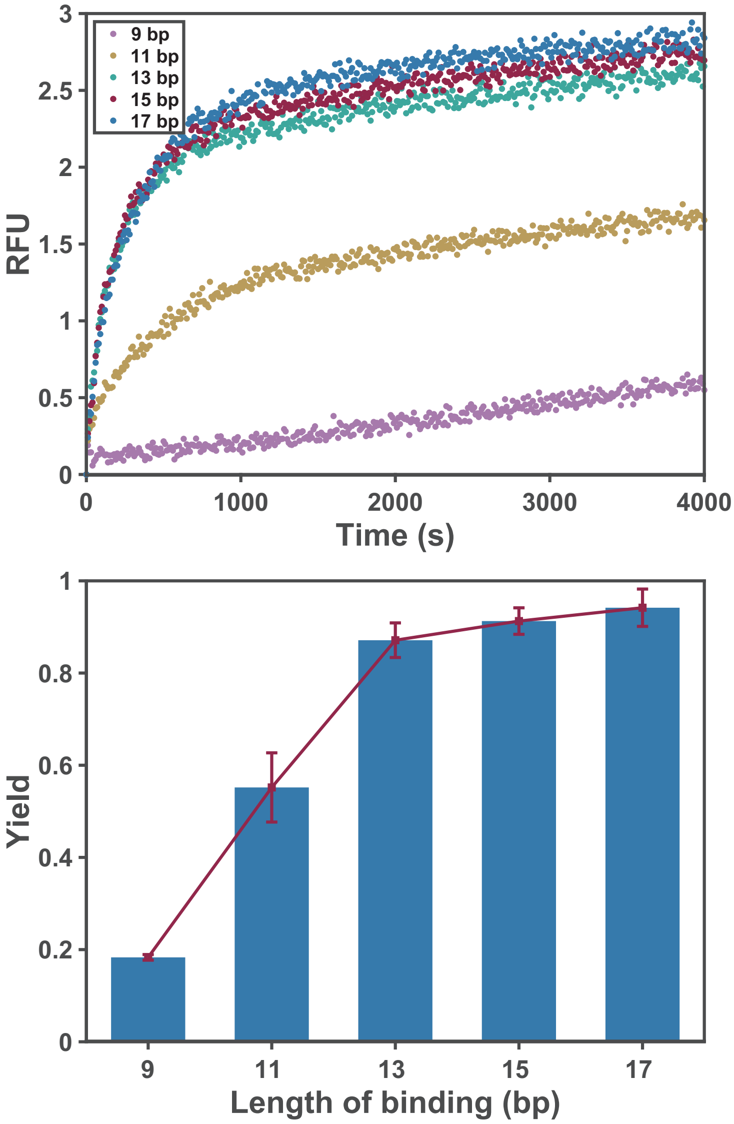

Figure. S 12: Optimizing the hybridization length between ssDNA and splitting modules. Each module possessed at least 13 bases that complementary to ssDNA to ensure a high yield. Error bars are presented as mean ± s.d., n = 3 independent experiments.

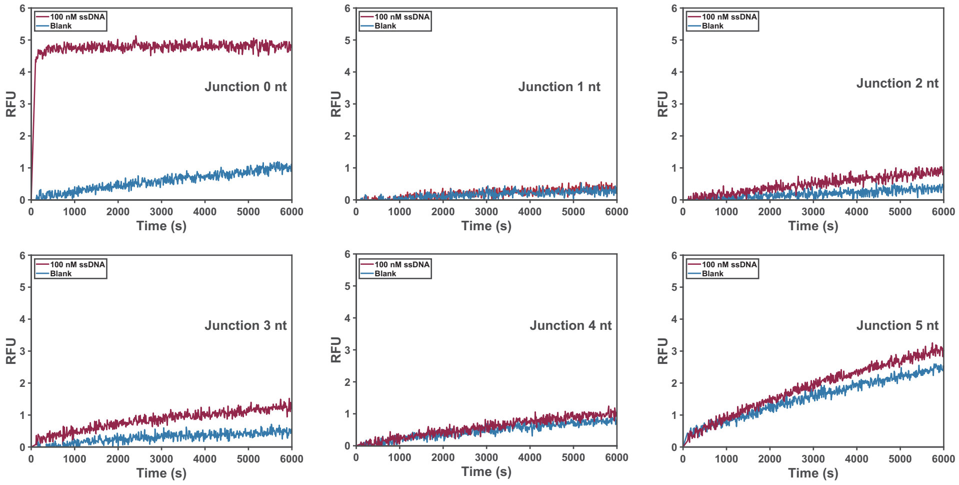

Figure. S 13: Optimizing length of junction between two modules. The junction significantly impedes the strand displacement reaction and increases leakage.This might be because we did not include the corresponding spacer in the substrate. We strategically eliminated junction between two modules in subsequent experiments.

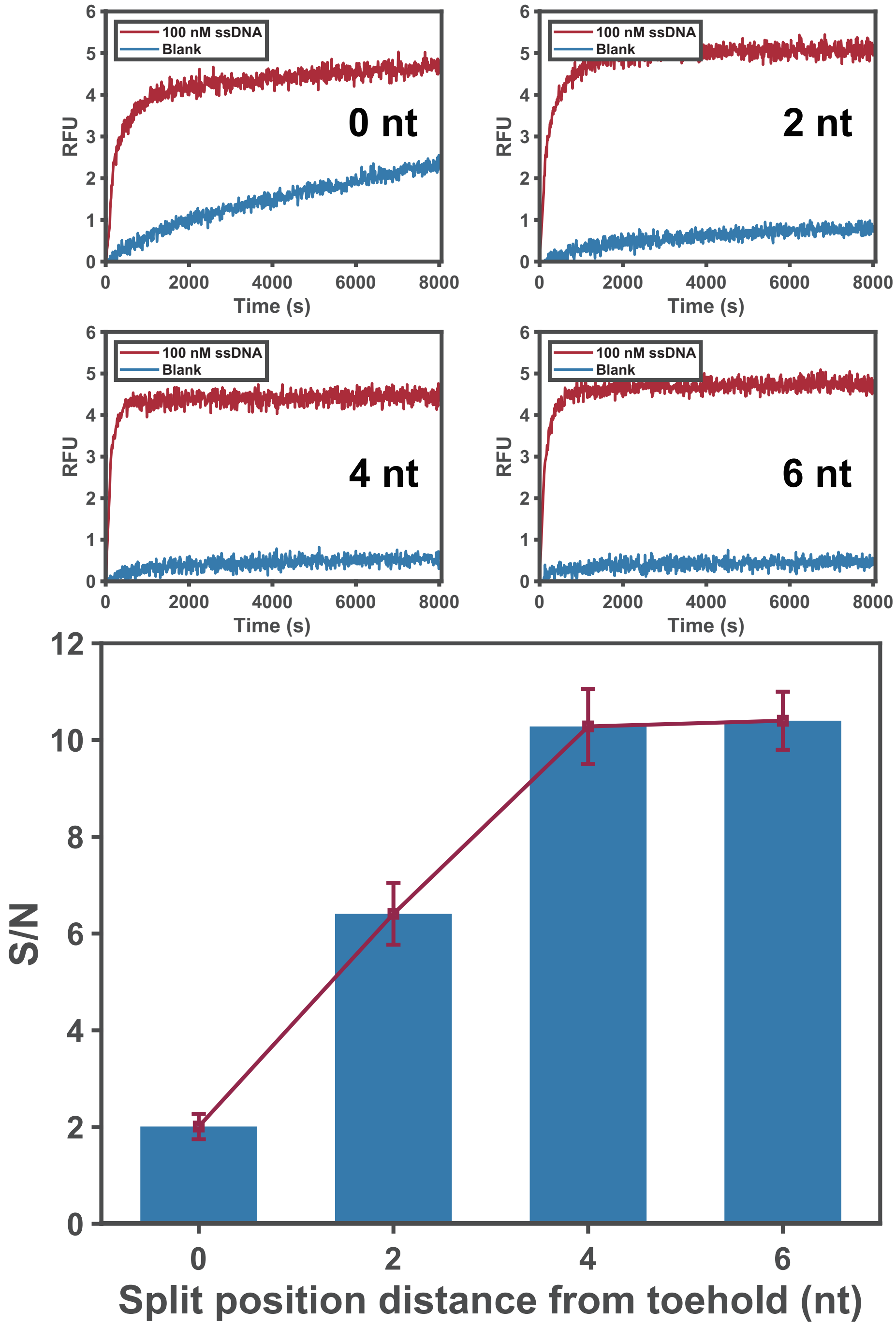

Figure. S 14: Optimizing split position distance from toehold to minimize leakage in the process of conversion.When the distance to the toehold exceeds 4 nt, a lower level of leakage is observed.. Error bars are presented as mean ± s.d., n = 3 independent experiments.

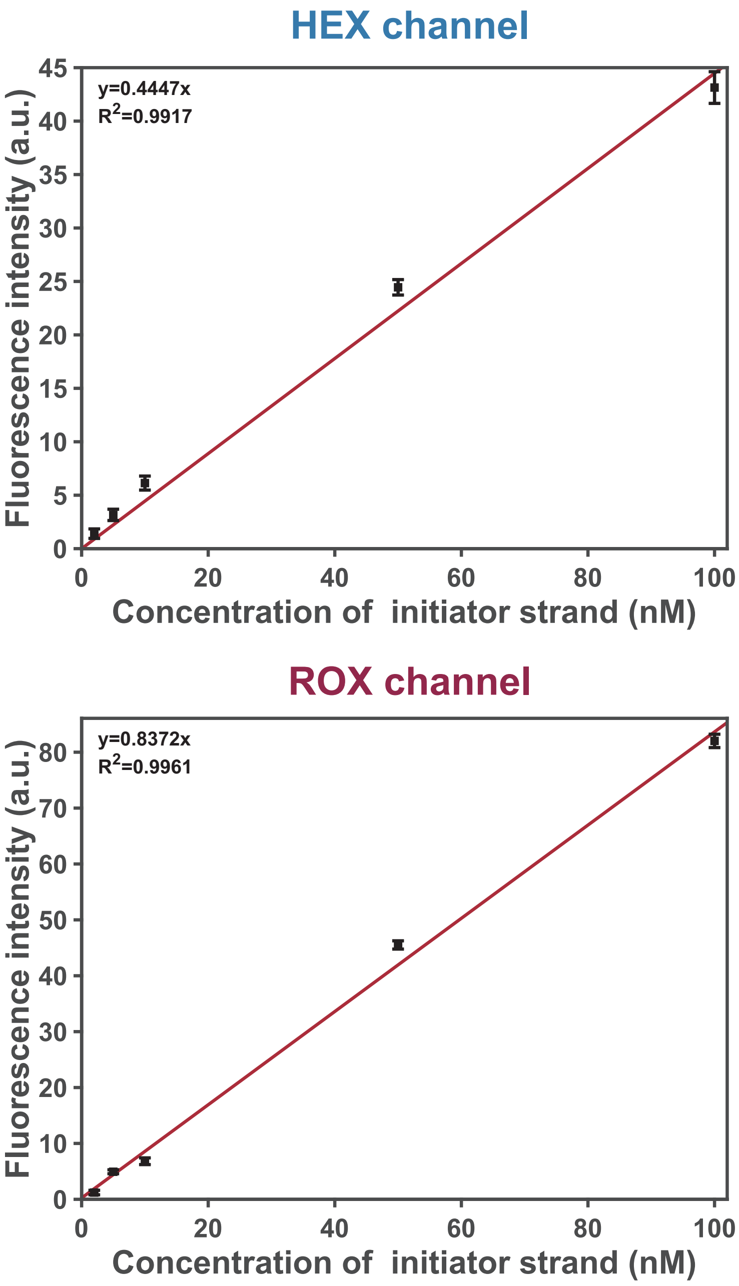

Figure. S 15: Standard curve of each reporter complex.

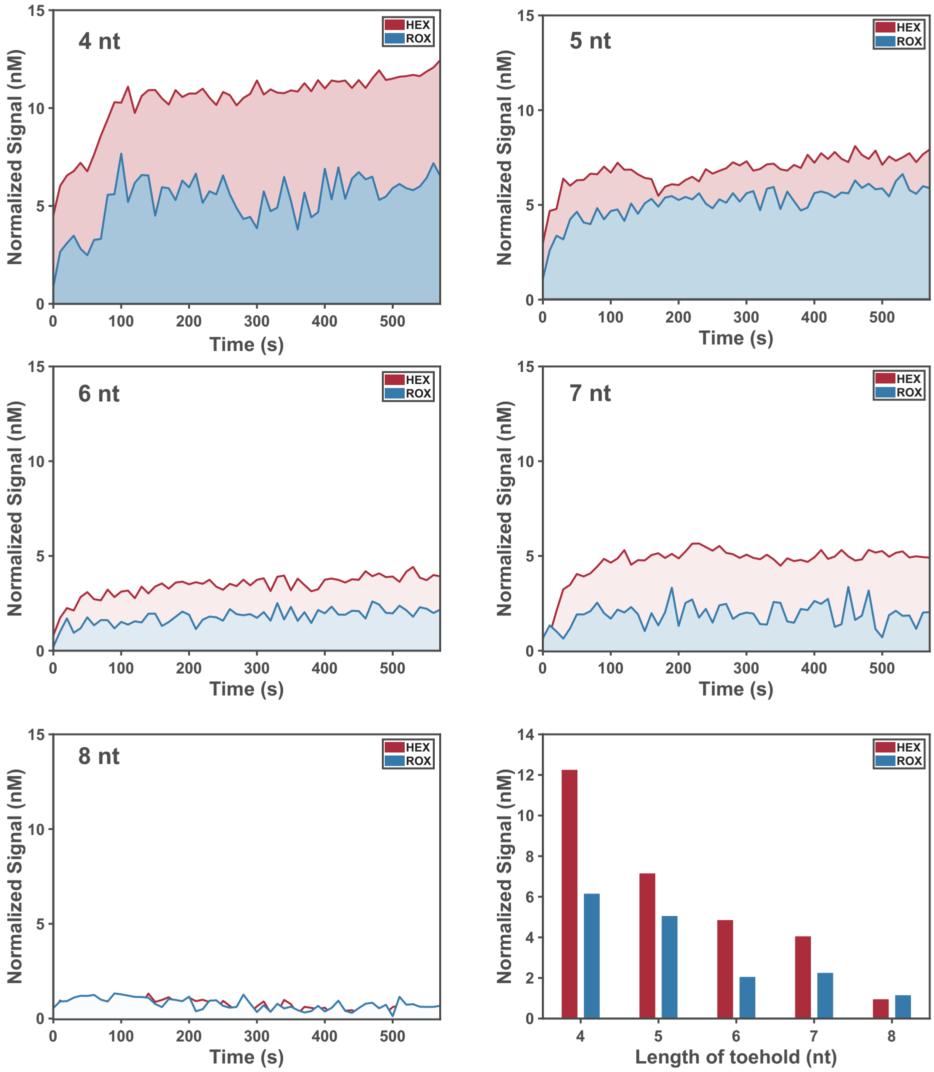

Figure. S 16: The annihilation efficiency is highly dependent on the length of toehold on the annihilator, toeholds with 8 nt have been intentionally designed to ensure the complete consumption of all minority species..

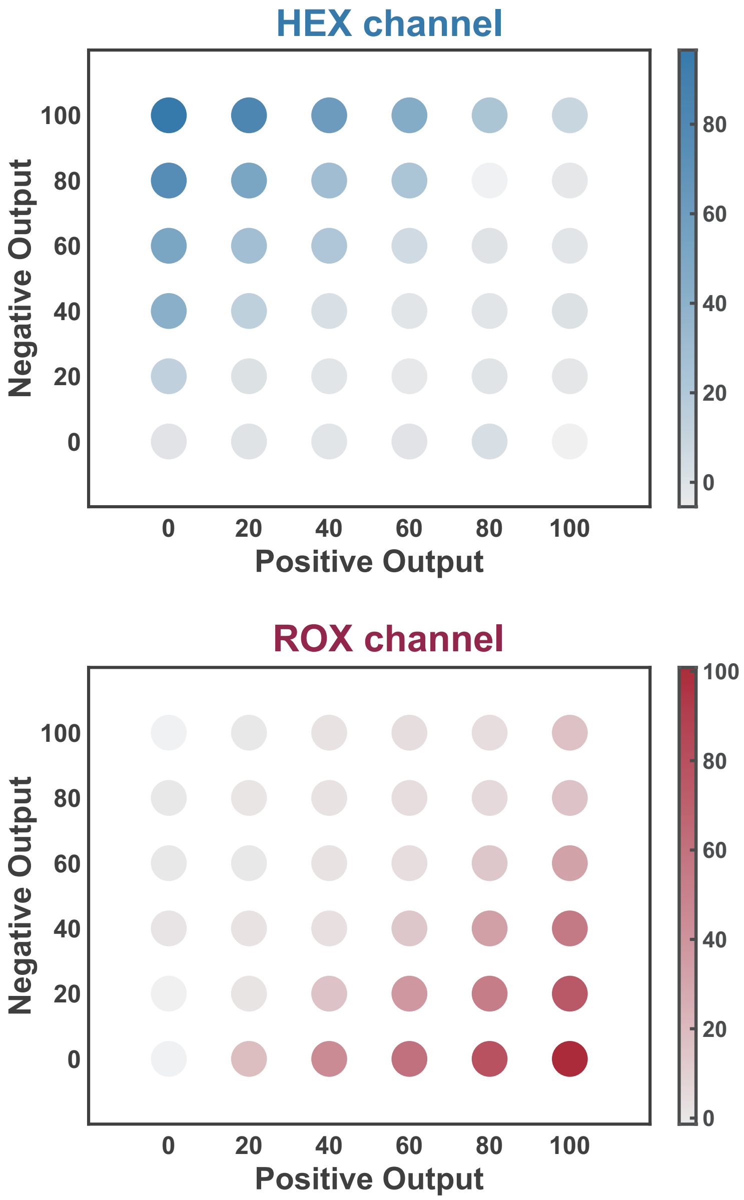

Figure. S 17: Performance of the optimized annihilator. A significant increase of fluorescence was observed in ROX channel only when the concentration of positive output surpassed that of negative output, while no fluorescence signal was detected in the HEX channel, and vice versa.

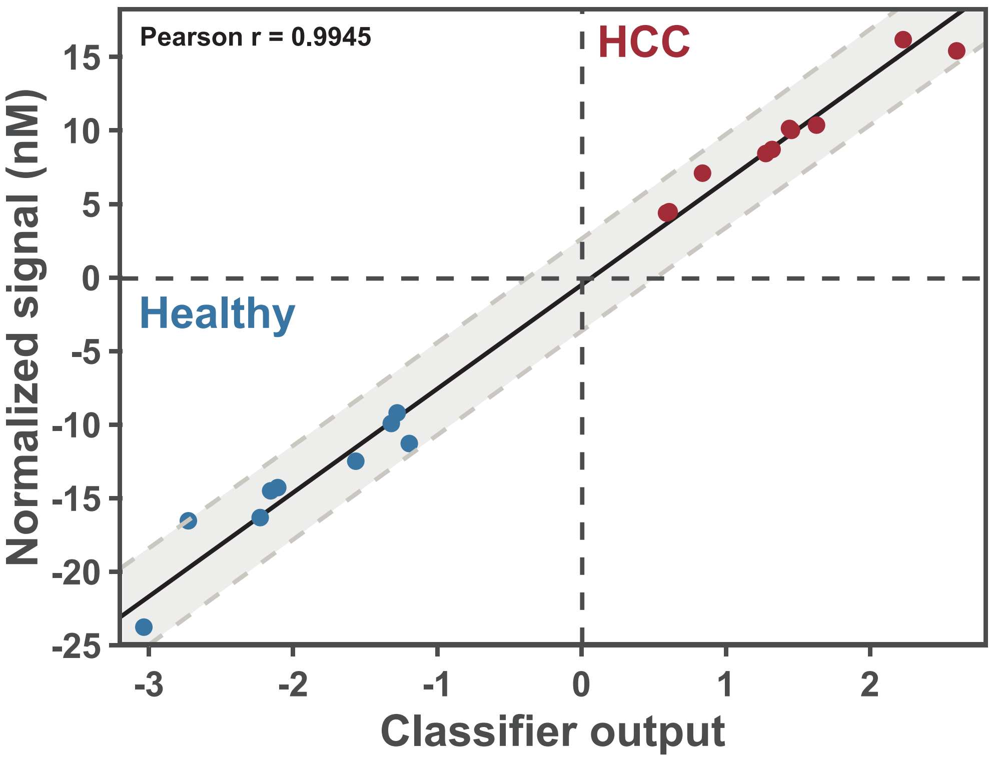

Figure. S 18: Correlation between the normalized signal intensity and the corresponding classifier output estimated in silico for each sample.

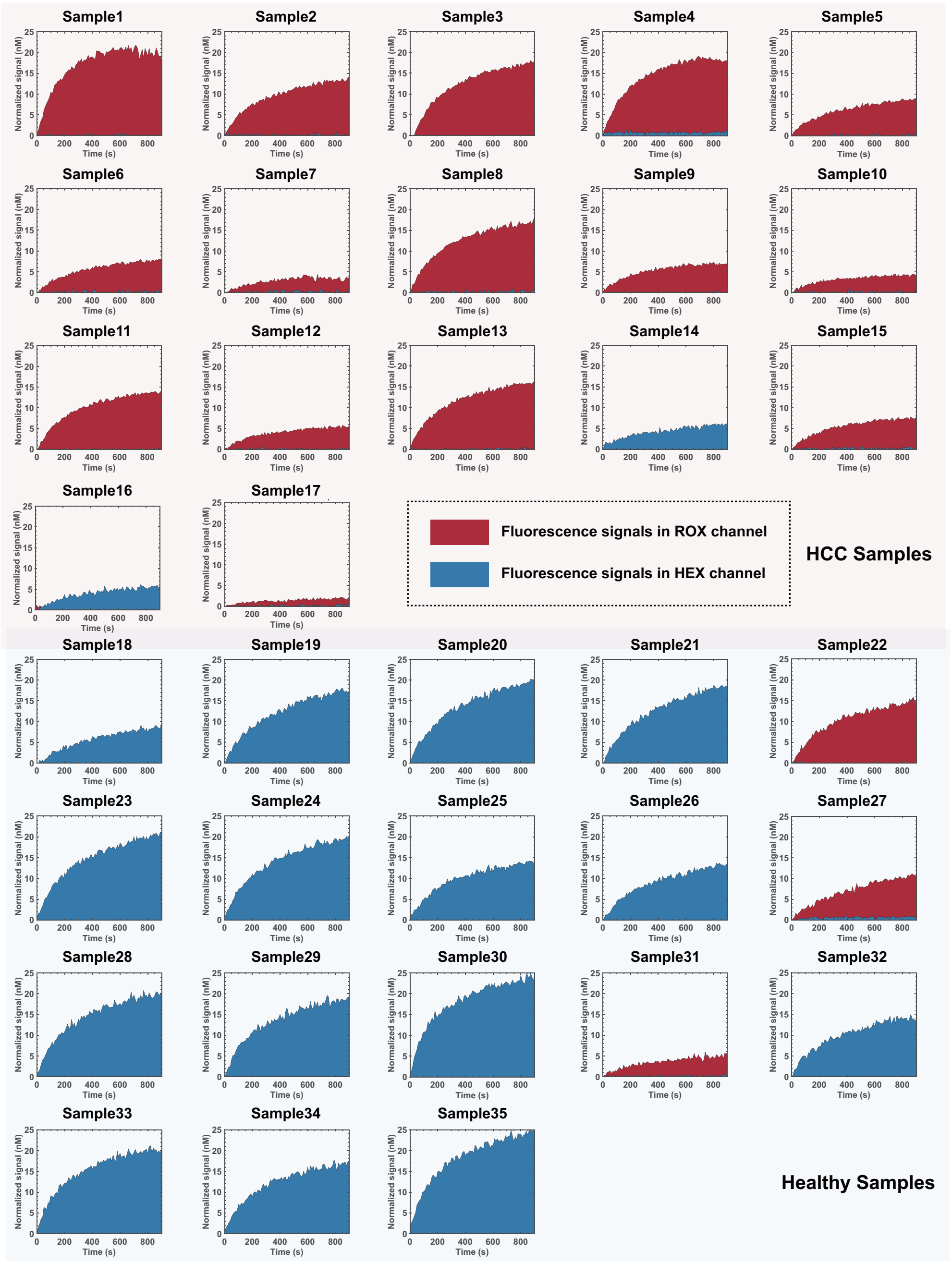

Figure. S 19: Profiling results for 17 patients with HCC and 18 healthy individuals.

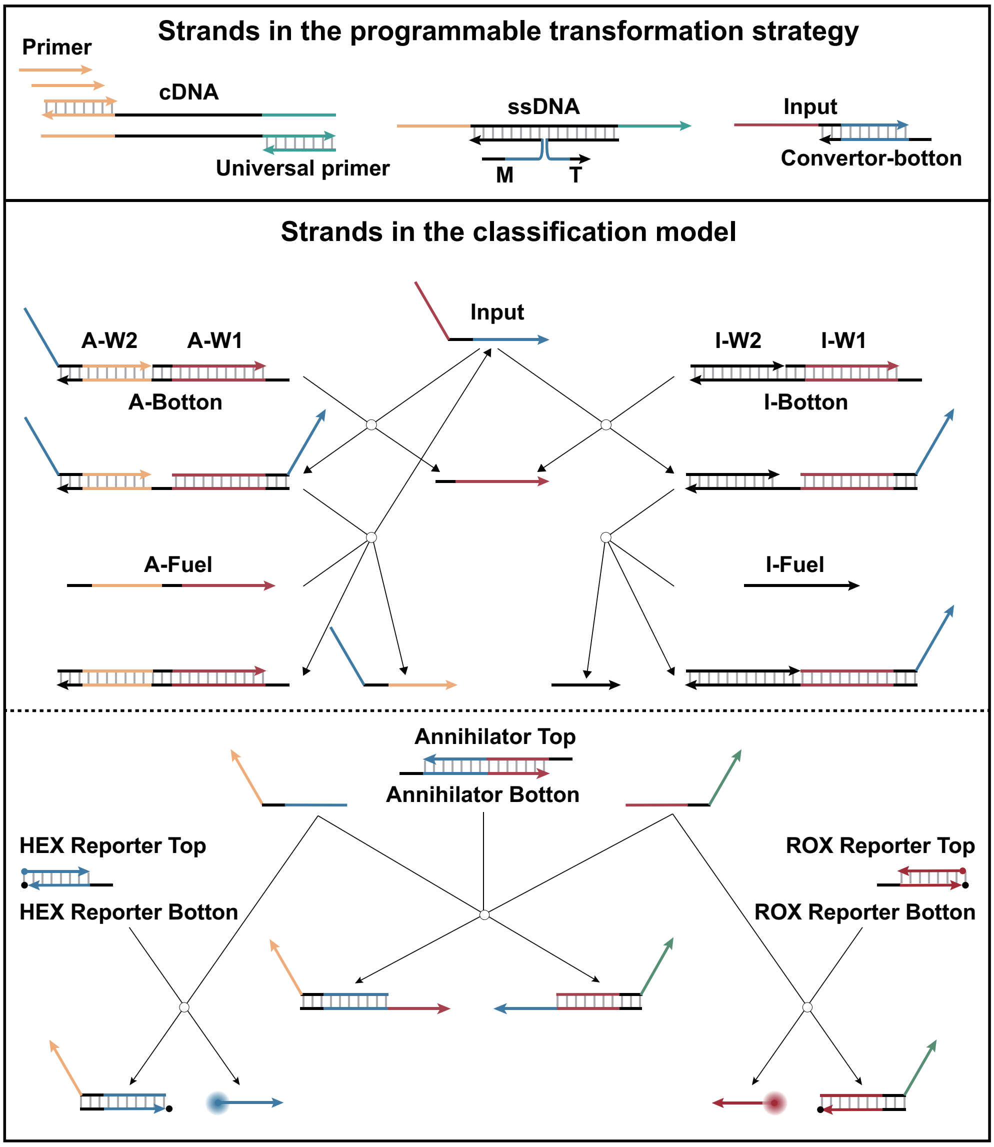

Figure. S 20: Strand name for the sequences used in the transformation strategy and classification model.

Table. S 1: Sequences of synthetic miRNA and corresponding primer

Name	Sequence (5' to 3')
miR-21	UAGCUUAUCAGACUGAUGUUGA
miR-21 primer	TAGCTTATCAGACTGATGTT
miR-221-3P	AGCUACAUUGUCUGCUGGGUUUC
miR-221-3P primer	AGCTACATTGTCTGCTGGGT
miR-1203	CCCGGAGCCAGGAUGCAGCUC
miR-1203 primer	CCCGGAGCCAGGATGCAGC
miR-1290	UGGAUUUUUGGAUCAGGGA
miR-1290 primer	TGGATTTTTGGATCAGGGA
miR-4258	CCCCGCCACCGCCUUGG
miR-4258 primer	CCCCGCCACCGCCTTGG
miR-4730	CUGGCGGAGCCCAUUCCAUGCCA
miR-4730 primer	CTGGCGGAGCCCATTCCATG

Table. S 2: Sequences of strands in programmable transformation strategy

**Name**	**Sequence (5' to 3')**
miR-93	CAAAGUGCUGUUCGUGCAGGUAG
miR-93 primer	CAAAGTGCTGTTCGTGCAGG
miR-93 ssDNA-34nt	CAAAGTGCTGTTCGTGCAGGTAGAAAAAAA AAAA
miR-93 ssDNA-30nt	CAAAGTGCTGTTCGTGCAGGTAGAAAAAAA AA
miR-93 ssDNA-26nt	CAAAGTGCTGTTCGTGCAGGTAGAAAAAAA
miR-93 ssDNA-22nt	CAAAGTGCTGTTCGTGCAGGTAGAAAAA
miR-93 ssDNA-18nt	CAAAGTGCTGTTCGTGCAGGTAGAAA
miR-93 M-J0	CGTCTCCAACACCTACCACGAACAGCACTT TG
miR-93 T-J0	TTTTTTTTTTTCTACCTGACCTAC
miR-93 M-J1	CGTCTCCAACACCTACCCACGAACAGCAC TTTG
miR-93 T-J1	TTTTTTTTTTTCTACCTGGACCTAC
miR-93 M-J2	CGTCTCCAACACCTACGCCACGAACAGC ACTTTG
miR-93 T-J2	TTTTTTTTTTTCTACCTGGCACCTAC
miR-93 M-J3	CGTCTCCAACACCTACGGCCACGAACAGC ACTTTG
miR-93 T-J3	TTTTTTTTTTTCTACCTGGCCACCTAC
miR-93 M-J4	CGTCTCCAACACCTACGTGCCACGAACAG CACTTTG
miR-93 T-J4	TTTTTTTTTTTCTACCTGGCACACCTAC
miR-93 M-J5	CGTCTCCAACACCTACGCTGCCACGAACA GCACTTTG
miR-93 T-J5	TTTTTTTTTTTCTACCTGGCAGCACCTAC

Table S2 continued

**name**	**Sequence (5' to 3')**
miR-93 M-J0-2	CGTCTCCAACACCTCACGAACAGCACTTTG
miR-93 T-J0-2	TTTTTTTTTTTCTACCTGACACCTAC
miR-93 M-J0-4	CGTCTCCAACACCACGAACAGCACTTTG
miR-93 T-J0-4	TTTTTTTTTTTCTACCTGCTACACCTAC
miR-93 M-J0-6	CGTCTCCAACCACGAACAGCACTTTG
miR-93 T-J0-6	TTTTTTTTTTTCTACCTGACCTACACCTAC
miR-93 Input	CATTCAATACCC/iHEXdT/ACGTCTCCAA CACCTAC
miR-93 Convertor-botton	GTAGGTGTAGGTGTTGGAGACG -BHQ1
miR-221-3p M	ACAGTACCATCAGCAGACAATGTAGCT
miR-221-3p T	TTTTTTTTTTTTGAAACCGAACCTCTCCAC
miR-221-3p Input	ACTACCAATCTACCCTACCATGAACCT
miR-221-3p Convertor-botton	GTGGAGAGGTTCATGGTAGGGT
miR-1203 M	TAAGTCCTAGGCATCCTGGCTCCGGG
miR-1203 T	TTTTTTTTTTTTGAGCTCTACCCCACATC
miR-1203 Input	CAATACTTCCCTAACTCCTAGCTACCC
miR-1203 Convertor-botton	GATGTGGGGTAGCTAGGACTTA
miR-1290 M	CCACAGCCTCTCTGATCCAAAAATCCA
miR-1290 T	TTTTTTTTTTTTTTCCAAACCTCTCCAC
miR-1290 Input	TTCTACTACACCACAGCCTCTAAACCT
miR-1290 Convertor-botton	GTGGAGAGGTTTAGAGGCTGTG

Table S2 continued

**name**	**Sequence (5' to 3')**
miR-4258 M	AACATCCACGTAGGCGGTGGCGGGG
miR-4258 T	TTTTTTTTTTTTCCACACTCTCAACC
miR-4258 Input	CCCTTCTAACTAACATCCACGTCACTC
miR-4258 Convertor-botton	GGTTGAGAGTGACGTGGATGTT
miR-4730 M ts	CGTCTCCAACAGGAATGGGCTCCGCCAG
miR-4730 T	TTTTTTTTTTTTGGCATCCTACACCTAC
miR-4730 Input	CATTCAATACCCTACGTCTCCAACACCTAC
miR-4730 Convertor-botton	GTAGGTGTAGGTGTTGGAGACG

Table. S 3: Sequences of strands in classification model

**Name**	**Sequence (5' to 3')**
miR-221-3p A-W1	CTCCGACTACCAATCTACCC
miR-221-3p A-W2	CTTTCCTACACCTACGTCTCCATCTAACTCACA
miR-221-3p A-Botton	CATGGTAGGGTAGATTGGTAGTCGGAGTGTGA GTTAGATGGAGACGTAGG
miR-221-3p A-Fuel	CCTACGTCTCCATCTAACTCACACTCCGACTA CCAATCTACCC
miR-221-3p I-W1	CTCCGACTACCAATCTACCC
miR-221-3p I-W2	TTATGCCATTCTCTCACCAATCC
miR-221-3p I-Botton	CATGGTAGGGTAGATTGGTAGTCGGAGGGATT GGTGAGAGAATGGCATAA
miR-221-3p I-Fuel	TTATGCCATTCTCTCACCAATCCCTCCG
miR-1203 A-W1	CCTCGCAATACTTCCCTAAC
miR-1203 A-W2	CATCTTTACCACCTCCTCCTGATACAGTTCACA
miR-1203 A-Botton	GCTAGGAGTTAGGGAAGTATTGCGAGGTGTGA ACTGTATCAGGAGGAGGT
miR-1203 A-Fuel	ACCTCCTCCTGATACAGTTCACACCTCGCAAT ACTTCCCTAAC
miR-1203 I-W1	CCTCGCAATACTTCCCTAAC
miR-1203 I-W2	CCGATCCTTCTTGAAACAACCCT
miR-1203 I-Botton	GCTAGGAGTTAGGGAAGTATTGCGAGGAGGGT TGTTTCAAGAAGGATCGG
miR-1203 I-Fuel	CCGATCCTTCTTGAAACAACCCTCCTCG
miR-1290 A-W1	CTGCCTTCTACTACACCACA
miR-1290 A-W2	CTTTCCTACACCTACGTCCTGAAACTTCCTACA
miR-1290 A-Botton	TAGAGGCTGTGGTGTAGTAGAAGGCAGTGTAG GAAGTTTCAGGACGTAGG

Table S3 continued

**name**	**Sequence (5' to 3')**
miR-1290 A-Fuel	CCTACGTCCTGAAACTTCCTACACTGCCTTCT ACTACACCACA
miR-1290 I-W1	CTGCCTTCTACTACACCACA
miR-1290 I-W2	GCTTGTAACTCAACACCACCTTC
miR-1290 I-Botton	TAGAGGCTGTGGTGTAGTAGAAGGCAGGAAGG TGGTGTTGAGTTACAAGC
miR-1290 I-Fuel	GCTTGTAACTCAACACCACCTTCCTGCC
miR-4258 A-W1	TCCGCCCCTTCTAACTAACA
miR-4258 A-W2	CTTTCCTACACCTACGTCCTGACTCTCAATCAA
miR-4258 A-Botton	ACGTGGATGTTAGTTAGAAGGGGCGGATTGAT TGAGAGTCAGGACGTAGG
miR-4258 A-Fuel	CCTACGTCCTGACTCTCAATCAATCCGCCCCT TCTAACTAACA
miR-4258 I-W1	TCCGCCCCTTCTAACTAACA
miR-4258 I-W2	CCAATAATTCCACCGGACTCCTT
miR-4258 I-Botton	ACGTGGATGTTAGTTAGAAGGGGCGGAAAGGA GTCCGGTGGAATTATTGG
miR-4258 I-Fuel	CCAATAATTCCACCGGACTCCTTTCCGC
miR-4730 A-W1	GCCCTCATTCAATACCCTAC
miR-4730 A-W2	CATCTTTACCACCTCCTCTCCAACTAACTTACG
miR-4730 A-Botton	TGGAGACGTAGGGTATTGAATGAGGGCCGTAA GTTAGTTGGAGAGGAGGT
miR-4730 A-Fuel	ACCTCCTCTCCAACTAACTTACGGCCCTCATT CAATACCCTAC
miR-4730 I-W1	GCCCTCATTCAATACCCTAC
miR-4730 I-W2	CTACTCATGCATACTTCACCTTC

Table S3 continued

name	Sequence (5' to 3')
miR-4730 I-Botton	TGGAGACGTAGGGTATTGAATGAGGGCGAAGG TGAAGTATGCATGAGTAG
miR-4730 I-Fuel	CTACTCATGCATACTTCACCTTCGCCCT
Annihilator Top 4nt	TCAGGAGGAGGTGGTAAAGATGCTTTCCTACACCTACGTC
Annihilator Botton 4nt	TGGAGACGTAGGTGTAGGAAAGCATCTTTACCACCTCCTC
Annihilator Top 5nt	TCAGGAGGAGGTGGTAAAGATGCTTTCCTACACCTACGT
Annihilator Botton 5nt	TGGAGACGTAGGTGTAGGAAAGCATCTTTACCACCTCCT
Annihilator Top 6nt	TCAGGAGGAGGTGGTAAAGATGCTTTCCTACACCTACG
Annihilator Botton 6nt	TGGAGACGTAGGTGTAGGAAAGCATCTTTACCACCTCC
Annihilator Top 7nt	TCAGGAGGAGGTGGTAAAGATGCTTTCCTACACCTAC
Annihilator Botton 7nt	TGGAGACGTAGGTGTAGGAAAGCATCTTTACCACCTC
Annihilator Top 8nt	TCAGGAGGAGGTGGTAAAGATGCTTTCCTACACCTA
Annihilator Botton 8nt	TGGAGACGTAGGTGTAGGAAAGCATCTTTACCACCT
HEX Reporter Top	HEX- CATCTTTACCACCTCC
HEX Reporter Botton	TCAGGAGGAGGTGGTAAAGATG -BHQ1
ROX Reporter Top	HEX- CTTTCCTACACCTACG
ROX Reporter Botton	TCAGGACGTAGGTGTAGGAAAG -BHQ2
